# Post‐Translational Modifications in Animal Circadian Clocks

**DOI:** 10.1002/advs.202521751

**Published:** 2026-02-09

**Authors:** Xianhui Liu, Yong Zhang

**Affiliations:** ^1^ Cambridge‐Suda Genomic Resource Center, The Fourth Affiliated Hospital, Suzhou Medical College Soochow University Suzhou Jiangsu China; ^2^ Key Laboratory of Endemic and Ethnic Diseases, School of Basic Medical Science, Ministry of Education &Engineering Research Center for Molecular Medicine Guizhou Medical University Guiyang China

**Keywords:** circadian clock, *Drosophila*, mammals, molecular oscillator, post‐translational modifications

## Abstract

The circadian clock is a fundamental biological system that synchronizes behavioral and physiological processes such as metabolism and immunity with the 24 h day‐night cycle. Disruption of circadian rhythms, often caused by modern lifestyle factors like shift work and jet lag, is closely associated with metabolic and mental disorders. In both mammals and *Drosophila*, the molecular oscillator consists of conserved transcriptional‐translational feedback loops (TTFLs) involving positive and negative regulatory elements that generate rhythmic gene expression. Post‐translational modifications (PTMs) of clock proteins play crucial roles in modulating the circadian period length, robustness, and responsiveness to environmental cues. Importantly, casein kinase 1 family‐dependent phosphorylation on both positive and negative elements in animal clocks highlights the evolutionary convergence of circadian timekeeping across species. This review focuses on PTMs and related mechanisms in circadian timekeeping and their roles in adapting to environmental and physiological signals in animals.

## Graphical abstract

Circadian clocks coordinate physiology with daily environmental cycles through conserved transcriptional–translational feedback loops. This review summarizes how post‐translational modifications fine‐tune clock function, highlights the evolutionary convergence of circadian timekeeping in Drosophila and mammals, and emphasizes the central of these modifications in enabling adaptability to metabolic, neural, and environmental challenges.

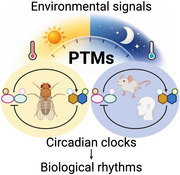

## 1 Introduction

The circadian clock is an essential mechanism that coordinates behavioral and processes, including metabolism, immunity, development, reproduction, to anticipate and respond to the environmental signals within a 24 h day‐night cycle [1, 2]. Disruption of the circadian clock tightly associates with metabolic diseases, such as cancer, type 2 diabetes, and cardiovascular diseases [3, 4, 5, 6], and mental disorders, such as Alzheimer's disease, seasonal affective disorders and anxiety disorders [7, 8, 9, 10]. In modern society, circadian disruption caused by misalignment of human circadian clock and environmental cues is common due to erratic lifestyle, shift work, and jet lag [3, 4, 5, 6, 7, 8, 9, 10]. Therefore, understanding the molecular mechanisms that regulate the oscillation of circadian clock is essential for designing therapeutic strategies to alleviate the deleterious impacts of circadian disruption and facilitate the interventions of diseases.

The circadian timing system is hierarchically organized in animals. In mammals, the master clock is localized in the suprachiasmatic nuclei (SCN) within the hypothalamus region, which is mainly entrained by the environmental light signals, the primary zeitgeber (time giver) [11, 12, 13]. The SCN communicates time information to the peripheral clocks present in all organs across the body, including the heart, lungs, liver, muscle, adipose tissue, and kidneys [14]. SCN controlled daily oscillation of humoral signals, body temperature and feeding/fasting cycles set the pace of peripheral clocks to align daily biological rhythms to light/dark cycles; notably, feeding/fasting cycles can also reset and synchronize the peripheral clocks independently of the master clock [15, 16]. Due to its genetic tractability and short lifespan, *Drosophila* has long served as a classical model organism in chronobiology. In recognition of their pioneering work elucidating the molecular mechanisms underlying circadian rhythms in *Drosophila*, Jeffrey C. Hall, Michael Rosbash, and Michael W. Young were awarded the 2017 Nobel Prize in Physiology or Medicine. In flies, the master clock is composed of ∼150 clock neurons in the dorsal‐lateral region of the brain, and peripheral clock exists in almost all tissues [1, 17, 18, 19].

**FIGURE 1:**
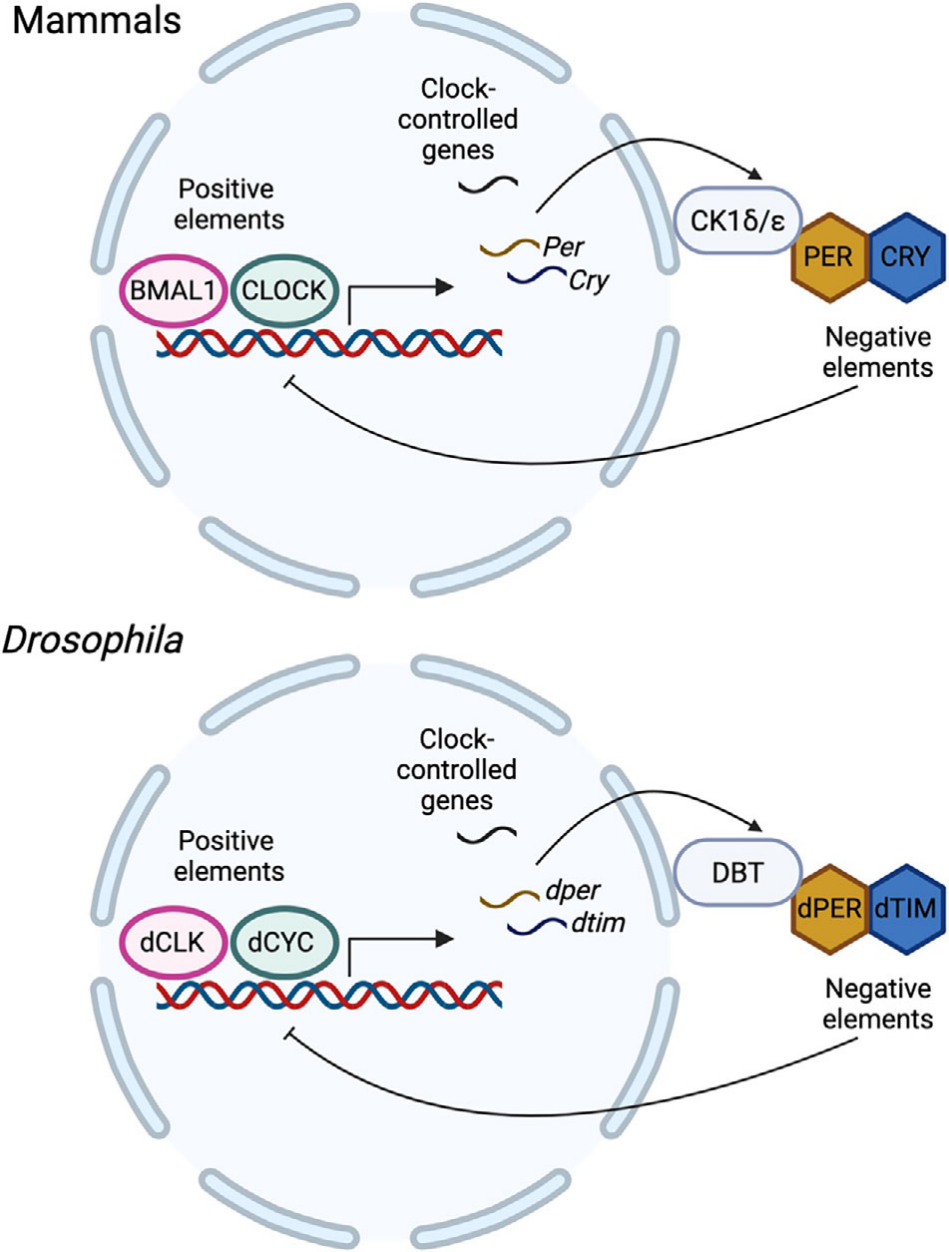
Molecular oscillators in mammals and *Drosophila*. Although the molecular components of the circadian oscillator are not entirely conserved between mammals and flies, the overall architecture of the transcriptional‐translational feedback loop (TTFL) is highly conserved. In this loop, positive elements activate the transcription of negative elements and clock‐controlled genes, whereas the negative elements feedback to repress their own expression. Created with Biorender.com. **Abbreviations**: BMAL1, brain and muscle ARNT‐like 1; CLOCK, circadian locomotor output cycles kaput; PER, period; CRY, cryptochrome; CK1δ/ε, casein kinase 1δ/ε; dCLK, *Drosophila* Clock; dCYC, *Drosophila* Cycle; dPER, *Drosophila* Period; dTIM, *Drosophila* Timeless; DBT, Doubletime.

In both the master and peripheral clocks, cell autonomous molecular oscillators consist of transcriptional‐translational feedback loops (TTFLs), which takes around 24 h for each cycle. Positive elements, such as *Drosophila* CLOCK (dCLK) and dCYCLE (dCYC) or Circadian locomotor output cycles kaput (CLOCK) and Brain and Muscle ARNT‐Like 1 (BMAL1) in mammals, binds to the E‐boxes in the promoters to drive the expression of negative elements: dPERIOD (dPER) and dTIMELESS (dTIM) in *Drosophila*, or PERIOD1‐3 (PER1‐3) and CRYPTOCHROME1‐2 (CRY1‐2) in mammals [20, 21, 22, 23] (Figure 1). Upon translation in the cytoplasm, these negative elements form repressor complexes, translocate into nucleus, and inhibit the activity of the positive elements [20, 21, 22, 23] (Figure 1). As the negative elements degrade, the subsequent cycle begins. Besides, the rhythmic expression of positive elements is controlled by auxiliary factors to enhance the robustness of the TTFL, including REV‐ERBs, Retinoid‐related orphan receptors (RORs), D‐box binding protein (DBP) and Nuclear factor interleukin 3 regulated (NFIL3) in mammals and Vrille (VRI) and Par domain protein1ε (PDP1ɛ) in flies [20, 21, 22, 23]

Post‐translational modifications (PTMs) of clock proteins are crucial for maintaining the 24 h circadian period. In the ancient cyanobacteria, for example, the biological timer is entirely dependent on 24 h phosphorylation rhythms of the KaiC protein [24]. In animals, the daily phosphorylation rhythm of dPER was first identified in 1994 [25]. Since then, PTMs have been shown to regulate the cellular localization, stability, and transcriptional activity of both positive and negative elements, playing an essential role in the phase shifting and modulating the clock in response to environmental cues‐including light, temperature, metabolism and other cellular signals. More importantly, while the clock proteins are not highly conserved among model organisms, recent studies highlighted that the function mode of kinases in casein kinase 1 (CK1) family is conserved among flies, mammals and non‐animal fungi (*Neurospora*), referring this is a common feature in animals [26]. In this review, we first summarize the PTM mechanisms in circadian timekeeping for both fly and mammalian models. We focused on the modifications of core clock proteins. Modifications of histone proteins and transcriptional machinery is beyond the scope of this review and is comprehensively reviewed by Takahashi group [20]. Second, we discuss PTMs as a mechanism by which animals respond to and adapt to the environmental and physiological signals, such as light, nutrition, temperature, and cellular stresses.

## 2 PTMs Within the Timekeeping Machinery

Despite proteins involved in animal TTFLs are not entirely conserved, the time‐of‐day features show high levels of similarity [20, 27, 28, 29, 30]. In midday, positive elements enter nucleus and promote the transcription of negative elements [27, 28]. At dusk, negative elements form repressor complex and enter the nucleus, and nuclear export delays nuclear accumulation of negative elements [29, 30, 31, 32, 33, 34]. At night, negative elements inhibit transcriptional activity of positive elements [27, 28, 31]. In late night to early morning, negative elements degrade, while positive elements degrade or become transcriptionally active for the subsequent cycle [35, 36, 37, 38, 39]. Nuclear export of positive elements has been shown to be necessary for degradation or replenishment [40, 41]. PTMs are essential modulators in all the processes above (Table 1 and Table 2).

**TABLE 1:** The Post‐translational modification (PTM) regulation of mammalian molecular oscillator.

Protein	Enzyme(s)	Function	Site	Modification	References
human Brain and muscle ARNT‐like 1 (hBMAL1)	Casein kinase 2α (CK2α)	Promote BMAL1 nuclear localization	S90	Phosphorylation	[47, 48]
	CK2β	Inhibit BMAL1 phosphorylation by CK2α	Uknown		[49]
	Uknown	Inhibits BMAL1 transcriptional activity	S78		[90]
	Mitogen‐activated protein kinase (MAPK)		S520		[95]
			T527		
			S592		
	CK1ε	Stimulates BMAL1 transcriptional activity	Uknown		[94]
	Glycogen synthase kinase 3β (GSK3β)	Facilitate BMAL1 ubiquitination	S17		[61]
			T21		
	Protein kinase Cγ (PKCγ)	Inhibit BMAL1 ubiquitination and stabilize BMAL1	Uknown		[258]
	CLOCK, TIP60, Sirtuin1 (SIRT1)	In activation phase, recruits BRD4‐P‐TEFb complex to promote elongation; in repression phase, facilitates the interaction between CRY1 and BMAL1/CLOCK complex	K537	Acetylation	[99, 100, 101, 102, 103, 104]
	Uknown	Facilitate BMAL1 ubiquitination	K259	SUMOylation	[65, 66]
	Ubiquitin‐specific protease2 (USP2)	Inhibit BMAL1 ubiquitination and stabilize BMAL1	Uknown	Ubiquitination	[64]
	O‐GlcNAc transferase (OGT)	Inhibit BMAL1 ubiquitination and stabilize BMAL1	Uknown	O‐GlcNAcylation	[69]
	Uknown	Inhibit BMAL1 ubiquitination and stabilize BMAL1	Uknown	S‐nitrosylation	[67]
human Circadian locomotor output cycles kaput (hCLOCK)	Uknown	Inhibits CLOCK nuclear localization and decrease the DNA binding of CLOCK	S38	Phosphorylation	[52]
			S42		
	CK2α	Disassemble CLOCK/BMAL1 complex and promote CLOCK nuclear export	S106		[53]
	PKCα/γ	Promote CLOCK nuclear localization	Uknown		[51]
	CK1δ	Inhibits BMAL1/CLOCK transcriptional activity	Uknown		[92]
	CK2		Uknown		
	GSK3β	Facilitate CLOCK degradation	S427		[62]
	Uknown		S431		
	Cyclin dependent kinase5 (CDK5)		T451		[63]
			T461		
	PKG2	Light response of CLOCK	Uknown		[243]
	mammalian Target of rapamycin (mTOR)‐AKT	Mediate the impact of osmotic stress on molecular oscillator	S845		[271, 272]
	OGT	Stabilize CLOCK	Uknown	O‐GlcNAcylation	[69]
	ZDHHC5		C194	S‐palmitoylation	[68]
human Period1 (hPER1)	CK1ε	Promote PER1 nuclear localization	S661	Phosphorylation	[123]
			S663		
	CK1α	Facilitate PER1 degradation	Uknown		[188]
hPER2	CK1δ	Promote PER2 nuclear localization	Uknown	Phosphorylation	[124, 127]
	GSK3β		Uknown		[125, 126]
	CDK5		S396		[128]
	CK1δ/ε	Reduce PER2 repressor activity	Uknown		[120]
		Facilitate PER2 degradation	S478		[37, 171, 173, 175]
		Stabilize PER2	S662		[166]
		Inhibit the phosphorylation of S478, and stabilize PER2	S665		[167, 168]
			S671		
			S674		
	Protein phosphatase1 (PP1)	Stabilize PER2	Uknown		[177]
		Inhibit PER2 nuclear localization	Uknown		[245]
	CK2	Enhances CK1ε‐dependent PER2 degradation, while stabilize PER2 by direct phosphorylation	Uknown		[186, 187]
	β‐transducin repeat‐containing protein1/2 (β‐TrCP1/2)	Facilitate PER2 degradation	Uknown	Ubiquitination	[171, 172, 173, 174]
	Mouse double minute2 (MDM2)		Uknown		[192]
	SIRT1		K680	Acetylation	[190, 191]
	OGT	Enhance repressor activity	S662	O‐GlcNAcylation	[206]
hPER3	CK1ε	Promote PER3 nuclear localization	Uknown	Phosphorylation	[119, 120, 121]
	CK1δ/ε	Enhance PER3 repressor activity	Uknown		[120]
human Cryptochrome1 (hCRY1)	AMP‐activated protein kinase (AMPK)	Enhance CRY1‐FBXL3 binding and facilitate CRY1 degradation	S71	Phosphorylation	[199]
			S280		
	F‐box and leucine‐rich repeat protein3 (FBXL3)	Facilitate CRY1 degradation	Uknown	Ubiquitination	[38, 39, 193]
	USP2a	Stabilize CRY1	Uknown		[196]
hCRY2	GSK3β	Facilitate CRY2 degradation	S554	Phosphorylation	[201, 202]
	MAPK, Dual‐specificity tyrosine‐regulated kinase 1A (DYRK1A)	Prime CRY2 S554 phosphorylation by GSK3β	S558		[202, 203]
	FBXL3	Facilitate CRY2 degradation	Uknown	Ubiquitination	[38, 39, 193]

**TABLE 2:** The PTM regulation of *Drosophila* molecular oscillator.

Protein	Enzyme(s)	Function	Site	Modification	References
*Drosophila* Clock (dCLK)	CK1α	decreases DNA binding activity of dCLK	S13	Phosphorylation	[89]
	Calcium/calmodulin‐dependent protein kinase II (CaMKII)	Light response of dCLK	Uknown		[235]
	CK2α	Stabilize dCLK	Uknown		[75]
	AMPKα		Uknown		[76]
	PP2A		Uknown		[74]
	USP8		Uknown	Ubiquitination	[73]
	Circadian trip (CTRIP)	Facilitate dCLK degradation	Uknown		[72]
dPER	MAPK	Promote SGG‐directed phosphorylation at S657	S661	Phosphorylation	[137, 138]
	Shaggy (SGG)	Promote dPER nuclear localization	S657		[138]
	CK2		S149		[132, 133, 134, 135]
			S151		
			S153		
	CK1α		Uknown		[136]
		Promotes DBT‐dependent PER phosphorylation and degradation	Uknown		
	PP2A	Promote dPER nuclear localization	Uknown		[139]
		Inhibit the phosphorylation of per‐short domain to promote dPER degradation	S610		[157]
			S613		
		Stabilize dPER	Uknown		[139, 157]
	NEMO	Inhibit the phosphorylation of S47, and stabilize dPER	S596		[35, 36, 114, 152, 153]
	Doubletime (DBT)		S585		
			S589		
			T583		
		Required for SLIMB recognition of dPER	S47		[35, 36]
		Inhibit dPER nuclear localization	Uknown		[130, 131]
		Light response	S826		[236]
			S828		
	Ribosomal S6 kinase (S6K)	Stabilize dPER	Uknown		[184, 185]
	Supernumerary limbs (SLIMB)	Facilitate dPER degradation	Uknown	Ubiquitination	[154, 155]
	OGT	Stabilize dPER	Uknown	O‐GlcNAcylation	[189]
		Inhibit dPER‐dCLK interaction and dPER repressor activity	S942		[207]
*Drosophila* Timeless (dTIM)	Phophatase of regenerating liver‐1 (PRL‐1)	Promote dTIM nuclear localization	S586	Phosphorylation	[147]
	PP1		Uknown		[148]
	SGG	Promote dTIM phosphorylation by CK2	S297		[146]
			S301		
	CK2	Promote the nuclear localization of dPER/dTIM complex	T305		
			S309		
			S313		
		Inhibit dTIM nuclear export	S1404		[30]
	JETLAG	Facilitate light‐induced dTIM degradation	Uknown	Ubiquitination	[224, 225]
	SLIMB	Facilitate dTIM degradation	Uknown		[155]
	CULLIN‐3	Facilitate dTIM degradation	Uknown		[233, 234]
dCRY	JETLAG	Facilitate light‐induced dCRY degradation	Uknown	Ubiquitination	[227, 228]
	BRUCE		Uknown		[229, 230]
	CG17735		Uknown		

### 2.1 The Regulation of Positive Elements

Unlike the daily oscillation in the protein abundance of negative elements, the protein levels of positive elements remain constant over a 24‐hour period. However, their phosphorylation level oscillates to generate the functional rhythms of positive elements. In 1998, dCLK was shown to be phosphorylated [42]. The transcriptional rhythms of dCLK is dispensable for the function of circadian clock [43], highlighting the importance of PTMs in the regulation of dCLK. The phosphorylation level of dCLK is tightly associated with its functional status: newly synthesized dCLK protein is hypophosphorylated in the cytoplasm; after import into the nucleus, dCLK becomes moderately phosphorylated and transcriptionally active. During repression phase, dCLK is exported from nucleus, hyperphosphorylated, and targeted for degradation [40]. In mammalian clock, BMAL1 and CLOCK also exhibit phosphorylation rhythms, with peak around dawn [44]. We devote this section to reviewing the evidence on the regulation of positive elements by PTMs, including phosphorylation, acetylation, SUMOylation, ubiquitination, O‐GlcNAcylation, S‐palmitoylation and S‐nitrosylation (Table 1 and Table 2).

#### 2.1.1 Cellular Localization

**FIGURE 2:**
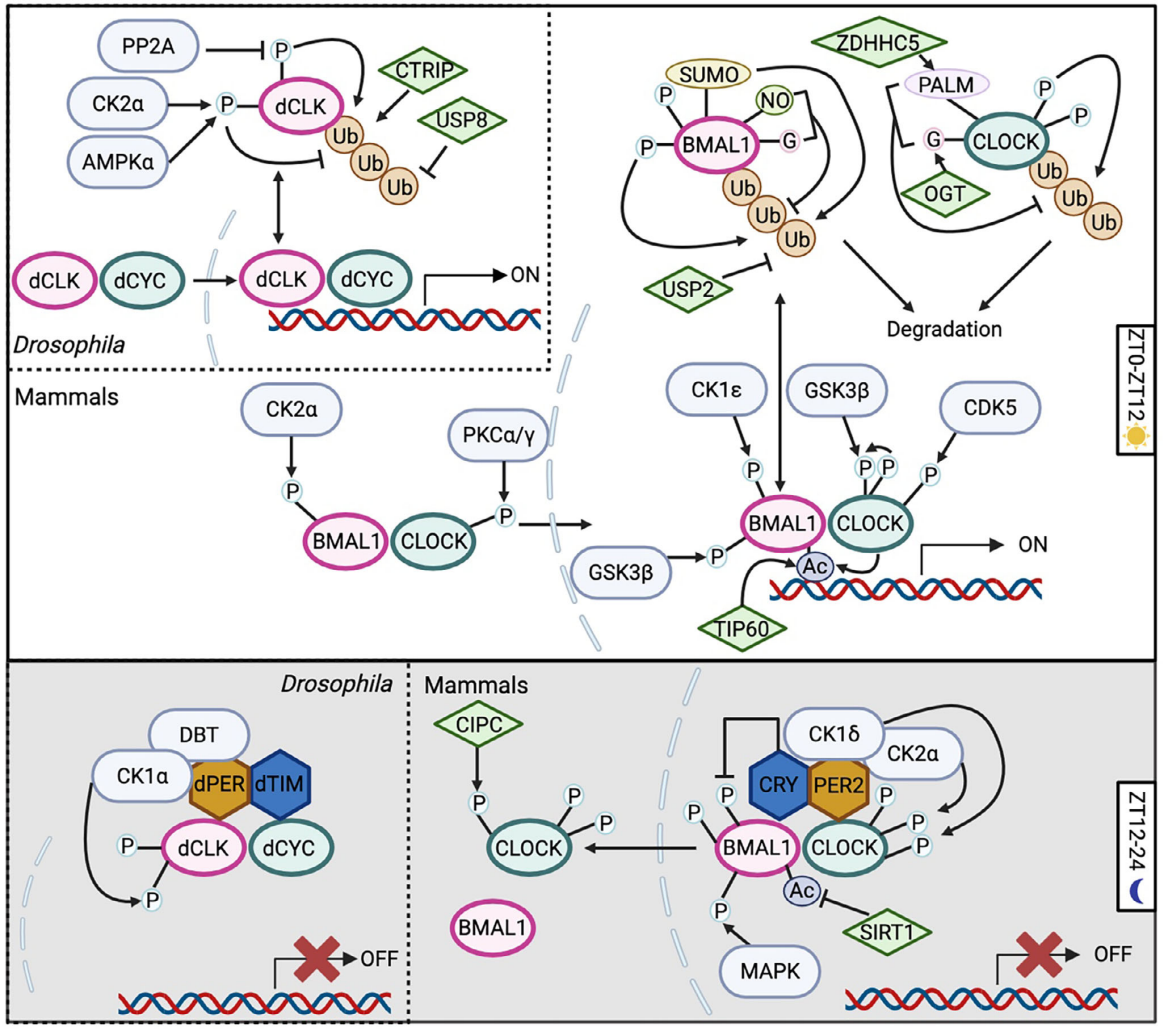
Post‐translational modification (PTM)–mediated regulation of positive elements in mammals and flies. (A) During the daytime (ZT0‐12) in mammals, newly synthesized BMAL1 and CLOCK translocate into the nucleus, facilitated by casein kinase 2α (CK2α) for BMAL1 and by protein kinase C α/γ (PKCα/γ) for CLOCK. In the nucleus, the degradation of these positive elements is coupled with their transcriptional activation. Glycogen synthase kinase 3β (GSK3β) and SUMOylation promote BMAL1 degradation through the ubiquitin–proteasome pathway, whereas ubiquitin‐specific protease 2 (USP2), S‐nitrosylation, and O‐GlcNAcylation stabilize BMAL1. For CLOCK, GSK3β and cyclin‐dependent kinase 5 (CDK5) trigger its degradation, while O‐GlcNAcylation and S‐palmitoylation counteract this process. During the night (ZT12‐24), the PER/CRY complex enters the nucleus and inhibits BMAL1/CLOCK activity. PER2 recruits CK1δ and CK2 to phosphorylate CLOCK, thereby displacing the BMAL1/CLOCK complex from DNA, whereas CRY attenuates BMAL1 function by suppressing its phosphorylation and destabilizing the heterodimer. In addition, mitogen‐activated protein kinase (MAPK)‐mediated phosphorylation of BMAL1 inhibits its activity. BMAL1 acetylation, regulated by CLOCK, TIP60, and SIRT1, exerts dual effects by recruiting distinct protein complexes during the day and night. In *Drosophila*, dCLK and dCYC form heterodimers that translocate into the nucleus. dCLK is stabilized through CK2α‐ and AMP‐activated protein kinase α (AMPKα)‐mediated phosphorylation and by the deubiquitinase USP8, whereas phosphorylation at sites targeted by Circadian trip (CTRIP) and protein phosphatase 2A (PP2A) promotes its degradation. At night, the dPER‐DBT complex brings in CK1α to phosphorylate dCLK and disrupt its DNA binding.

**FIGURE 3:**
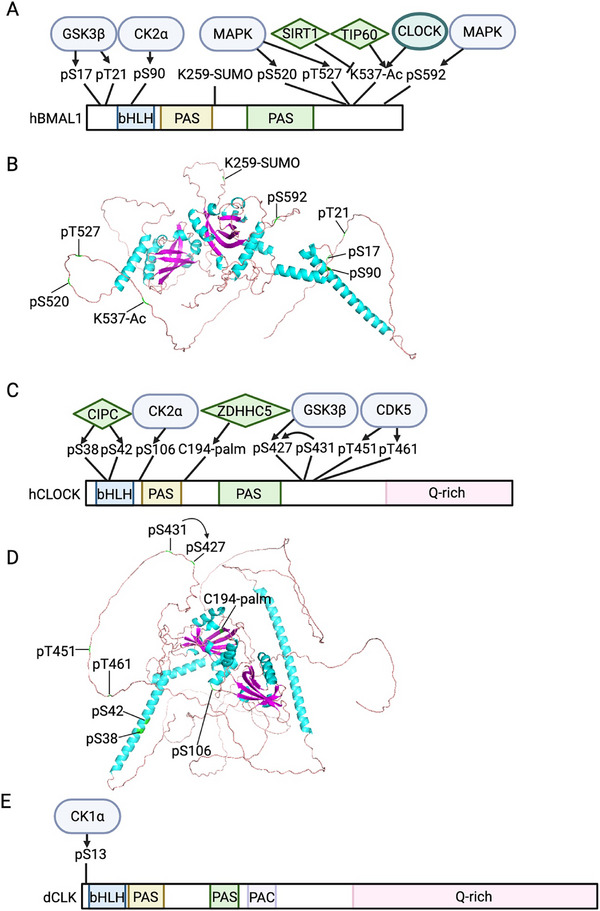
PTM landscape of positive elements in mouse and *Drosophila*. (A, C, E) Schematic representation of the functional modification sites on BMAL1 (A), CLOCK (C), and dCLK (E) as described in Figure 2. (A‐B) Structural model of BMAL1 highlighting the PTM sites in panel (A). (C‐D) Structural model of CLOCK highlighting the PTM sites in panel (C). Human BMAL1 and CLOCK are depicted due to their high sequence conservation with the murine homologs. Created with Biorender.com.

To start or terminate the oscillation of molecular clock, the temporal nuclear import and export of positive elements is essential, which is highly regulated by their phosphorylation and protein‐protein interaction (Figure 2 and Figure 3). In mouse liver, mBMAL1 is predominantly nuclear, as it contains two nucleus localization signal (NLS) sequence (N^36^RKRK and K^89^RRRR) [45, 46]. The Sasson‐Corsi group first demonstrated that Casein kinase 2α (CK2α) promotes hBMAL1 nuclear localization by phosphorylating it at S90 [47, 48], and thereby triggering the expression of downstream clock‐controlled genes [48]. Since CK2 usually function as a holoenzyme composed of CK2α and CK2β, it is intriguing to find that CK2β inhibits the phosphorylation of BMAL1 as opposite to CK2α in tissue culture system [47]. A follow‐up study clarified that CK2α phosphorylates BMAL1 as a monomer, while CK2β inhibits this modification [49]. Regarding the nuclear localization of mCLOCK, early studies suggested that mBMAL1 binds and shuttles mCLOCK into the nucleus through binding to mCLOCK [46, 50]. In a mechanistic study on clock resetting, serum shock of fibroblast cells was found to trigger CLOCK nuclear localization and a surge in *Per1* expression, regulated by Ca^2+^‐dependent PKCα and PKCγ‐directed CLOCK phosphorylation [51]. In mouse liver, phosphorylation of mCLOCK at S38 and S42 by unknown kinase(s) inhibits its nuclear localization, reducing its DNA‐binding and transactivation activity. S38 and S42 phosphorylation could be stimulated by the interaction between mCLOCK and Clock‐interacting proteins circadian (CIPC) [52].

The nuclear export of BMAL1/CLOCK complex is also regulated by phosphorylation. During the repression phase, CRY proteins inhibit the phospho‐occupancy of hBMAL1 S90, which promotes the nuclear export of mBMAL1 via its nuclear export signal sequence (NES, L^143^SDDELKHLIL, L^311^SCLVAIGRL, I^360^LAYLPQELL) [45, 46]. In contrast to its role in facilitating BMAL1 nuclear importation, CK2α phosphorylates CLOCK at S106, disassembling CLOCK/BMAL1 complex and increasing CLOCK‐Exportin1 binding for CLOCK nuclear export in hepatocellular carcinoma (HCC) cells [53].

Research on the regulation of dCLK subcellular localization is relatively limited. To date, only one study has shown that in S2 cells, dCYC interacts with dCLK through the PASA domain in cytoplasm, promoting the phosphorylation and nuclear import of dCLK [54]. In summary, CK2 is the major kinase that regulates BMAL1 subcellular localization, while the localization of CLOCK is regulated by multiple kinases, highlighting the control by several signaling pathways (Figure 2 and Figure 3, Table 1 and Table 2). However, in flies, it still remains to be investigated whether the phosphorylation status or other PTMs of dCLK impact its nuclear trafficking (i.e., import and export).

#### 2.1.2 Degradation Coupled With Transactivation

In addition to cellular localization, PTMs also regulate the transcriptional activity and stability of positive elements. Intriguingly and counterintuitively, the degradation of positive elements in molecular clocks is strongly coupled with their transactivation activity. This aligns with the “black widow model” first proposed in 2000s, where transcription factors, such as Jun, Myc, p53, Fos, and HIF1‐α, act as potent transcriptional activator, while phosphorylation marks them for ubiquitin‐directed proteasomal degradation [55, 56]. The “black widow model” provides an mechanism for preventing too much expression of downstream genes during transcriptional activation [55, 56]. In circadian clock, the phosphosites recognized by ubiquitin ligases are called phosphodegron. In mammalian clocks, the most direct evidence of the “black widow model” showed that MG132, a proteasome inhibitor, can compromise the transcription of the *Per1::Luc* reporter, while stabilizing CLOCK and BMAL1 [46]. In NIH3T3 cell lines, the expression of BMAL1/CLOCK immediate target genes requires a functional proteasome [57]. Additionally, the negative elements also utilize this mechanism to inhibit BMAL1/CLOCK function. CRY acts as a transcriptional repressor by inhibiting the phosphorylation of BMAL1 [46, 58], destabilizing the heterodimer [59], and/or stabilizing unphosphorylated BMAL1/CLOCK [60].

Multiple PTMs regulate BMAL1 and CLOCK stability (Figure 2 and Figure 3, Table 1). Glycogen synthase kinase 3β (GSK3β) has been shown to facilitate BMAL1 ubiquitination by phosphorylation at S17 and T21 [61]. Two kinases are known to trigger the degradation of CLOCK: GSK3β phosphorylates CLOCK at S427 with priming phosphorylation at S431 [62], while Cyclin‐dependent kinase 5 (CDK5) phosphorylates CLOCK at T451 and T461 [63]. Besides, Ubiquitin specific protease 2 (USP2) deubiquitinates BMAL1 and increases its stability [64]. Since USP2 mRNA and protein oscillate in mouse liver, in antiphase with the expression of BMAL1‐target genes [64], USP2 could regulate the time‐of‐day specific stability and activity of BMAL1. SUMOylation of BMAL1 also interacts with ubiquitination to regulate BMAL1 stability [65, 66]. Specifically, SUMOylation of BMAL1 at K259, a conserved site from *Drosophila* and zebra fish to mouse and human, facilitates its ubiquitination and degradation [65, 66]. Mutation of BMAL1 K259 reduces its transcriptional activity [66], which is consistent with the fact that SUMOylated BMAL1 is predominantly located in nuclear bodies associated with transcriptionally active chromatins [66]. Also, BMAL1 SUMOylation is rhythmic in mouse liver peaking during its activation phase [65, 66], which further supports the role of SUMOylation in BMAL1 stability and transactivation. These observations fit the “black widow model” of transcriptional activation. Finally, S‐nitrosylation of BMAL1 [67], S‐palmitoylation of CLOCK at C194 by ZDHHC5 [68] and O‐GlcNAcylation of both BMAL1 and CLOCK [69] have been shown to stabilize BMAL1 and CLOCK by inhibiting their ubiquitination. However, given O‐GlcNAcylation interacts with phosphorylation [70, 71], it remains to be determined whether O‐GlcNAcylation interacts with phosphorylation events that promotes ubiquitination. The ubiquitin ligases of BMAL1 and CLOCK also remain to be identified.

In *Drosophila*, CTRIP, a HECT‐domain E3 ubiquitin ligase encoded by *circadian trip*, is restrictedly expressed in clock neurons and destabilizes dCLK [72], while ubiquitin specific protease 8 (USP8) deubiquitinates dCLK [73]. There is evidence suggesting the existence of phosphodegron, as the phosphatase 2A complex with the WDB regulatory subunit can stabilize dCLK by decreasing its phosphorylation [74]. The two kinases that are shown to regulate dCLK stability, including CK2α[75] and AMP‐activated protein kinase α (AMPKα) [76], both stabilize dCLK by phosphorylation. Therefore, the kinase(s) and the phosphosite(s) that promote dCLK ubiquitination and degradation is still unknown.

In summary, the stability of positive elements in both mammals and flies is regulated by phosphorylation, ubiquitination and proteasome‐directed degradation, with several key kinases and USP proteins identified in both systems (Figure 2 and Figure 3, Table 1 and Table 2). However, since the stability of mammalian BMAL1/CLOCK is also modulated by various additional PTMs, including SUMOylation, O‐GlcNAcylation, S‐palmitoylation and S‐nitrosylation, it would be interesting to investigate whether these PTMs also regulate the stability of dCLK in *Drosophila*. More importantly, given that O‐GlcNAcylation [77, 78], S‐palmitoylation [79, 80] and S‐nitrosylation [81] are sensitive to metabolic state, future investigation are warranted to determine whether these PTMs represents a link between circadian clock and metabolism.

#### 2.1.3 The Regulation of Transcriptional Activity Independent of Degradation

As the molecular oscillator operates as a negative feedback loop, the function of positive elements is inhibited by negative elements. The inhibitory function of negative elements includes three modes: 1) Blocking by physical interaction between negative and positive elements on DNA, 2) Displacement by removing the positive elements from DNA, 3) Sequestration by preventing positive elements from binding to DNA [82, 83, 84]. Negative elements can displace and sequester positive elements by recruiting kinases to phosphorylate them (Figure 2 and Figure 3, Table 1 and Table 2). This regulatory paradigm was first proposed in *Drosophila*. In early studies, it was proposed that the repressor complex, dPER/dTIM, recruits DOUBLETIME (DBT) kinase to phosphorylate dCLK, which is correlated with decreased dCLK transcriptional activity and stability [85]. However, in *dbt^ar^*, a *dbt* null mutant, dCLK is hyperphosphorylated instead of hypophosphorylated [86]. This unexpected finding indicated that DBT kinase activity might not be required for dCLK phosphorylation. Intriguingly, in perΔ flies, which lack the DBT binding domain, dCLK remains hypophosphorylated and retained the ability to bind to the *dper* promoter [87], suggesting dPER/DBT complex could recruit other kinase(s) to phosphorylate dCLK and inhibit its function. Indeed, utilizing *dbt^ar^* flies, Yu et al. [88] demonstrated that dPER/DBT noncatalytic activity is essential for dCLK hyperphosphorylation and transcriptional repression. More recently, Chiu group showed that the dPER/DBT repressor complex recruits CK1α to downregulates dCLK transcriptional activity [89]. CK1α‐dependent phosphorylation near bHLH binding domain decreases DNA binding activity of dCLK. Interestingly, CK1α‐dependent CLK phosphorylation does not influence stability of dCLK, indicating the coupling of stability and transactivation can be phosphosite‐specific.

Similarly, in the mammalian clock, both BMAL1 and CLOCK are phosphorylated in the bHLH DNA binding domain (S78 in BMAL1 [90] and S38/S42 in CLOCK [52]). The phosphorylation of these residues reduces DNA binding and transcriptional activity of BMAL1 and CLOCK. In a more recent study, mathematical modeling predicted that the phosphorylation of DNA binding domains of BMAL1/CLOCK is essential for PER‐dependent inhibition (displacement), which is further supported by experimental evidence [91]. In terms of the kinases that are required for the repression phase, Cao et al. [92] found that PER2 recruits CK1δ and CK2 to hyperphosphorylate CLOCK and remove the BMAL1/CLOCK complex from DNA. Mathematical modeling showed that these repressive phosphorylations of BMAL1/CLOCK synergistically collaborate with other repression mechanisms to maintain the robustness of daily oscillation [93]. More importantly, the hyperphosphorylation of activators is a conserved feature from *Neurospora* to mammals that achieve repression even with fewer repressors, i.e., the low molecular ratio between repressors and activators [26]. However, whether CK1δ and CK2 mediate the phosphorylation of CLOCK S38/42 or other sites remains to be investigated.

There are also phosphorylation events of positive elements, independent of the negative elements (Figure 2 and Figure 3, Table 1 and Table 2). CK1ε phosphorylates BMAL1 and stimulates its transcriptional activity in HEK 293 cells [94]. Mass spectrometry analysis found that Mitogen‐activated protein kinase (MAPK) phosphorylates mBMAL1 at S527, T534 and S599, and inhibits BMAL1/CLOCK activity [95]. As MAPK activity is rhythmic and peaks at mid to late night when the levels of PERs and CRYs are low [96, 97, 98], it is likely that MAPK serves as an inhibitory mechanism during the derepression phase of TTFL.

Other than phosphorylation, the function of BMAL1 is also regulated by acetylation (Figure 2 and Figure 3, Table 1). In the 2000s, Sasson‐Corsi group found that CLOCK is a histone acetyltransferase (HAT) by protein structural comparison. CLOCK can acetylates mBMAL1 at lysine 537 (K537) in addition to histone H3 and H4 [99, 100]. However, this finding was challenged as BMAL1 is still acetylated in CLOCK‐deficient MEF cells, and TIP60, a lysine acetyltransferase found in BMAL1/CLOCK complex, has been shown to acetylate BMAL1 K537 [99, 101]. More importantly, acetylation of BMAL1 oscillates through a 24‐hr period with a peak expanding over the activation phase to repression phase in mouse liver (ZT9‐15) [100, 102]. In the activation phase, BMAL1 acetylation recruits BRD4‐P‐TEFb complex to release RNA Pol II from promoter‐proximal pause sites for productive elongation [101]. In the repression phase, studies have shown that BMAL1 acetylation facilitates the interaction between CRY1 and BMAL1/CLOCK complex, thereby inhibiting BMAL1 transcriptional activity [100, 103, 104]. To achieve the dynamic regulation of BMAL1 acetylation rhythm, a histone deacetylase (HDAC) protein is likely involved. Indeed, Sirtuin1 (SIRT1), A NAD^+^‐dependent HDAC, has been shown to constantly interact with CLOCK, and SIRT1 can deacetylate BMAL1 at K537 [102]. In the mouse liver, deacetylase activity of SIRT1 exhibits rhythmic pattern, with peak activity at the mid to late night, which coincides with the decline of BMAL1 acetylation [100, 102].

In summary, phosphorylation of DNA‐binding domains of positive elements, often mediated through repressor‐dependent kinase recruitment, emerges as a conserved mechanism from *Drosophila* to mammals that effectively reduces activator binding affinity and transcriptional output (Figure 2 and Figure 3, Table 1 and Table 2). Specifically in mammalian system, acetylation regulates BMAL1 function in a time‐of‐day specific manner (Figure 2 and Figure 3, Table 1). Nevertheless, it remains to be investigated whether the acetylation of positive elements is a conserved mechanism between flies and mammals. In flies, TIP60 complex component was found to interact with dCLK and is involved in circadian regulation. Whether TIP60 complex directly acetylates dCLK/CYC remains unclear [105, 106]. Moreover, how these PTMs are integrated to carry out the transcriptional activity of positive elements requires future studies.

### 2.2 The Regulation of Negative Elements

**FIGURE 4:**
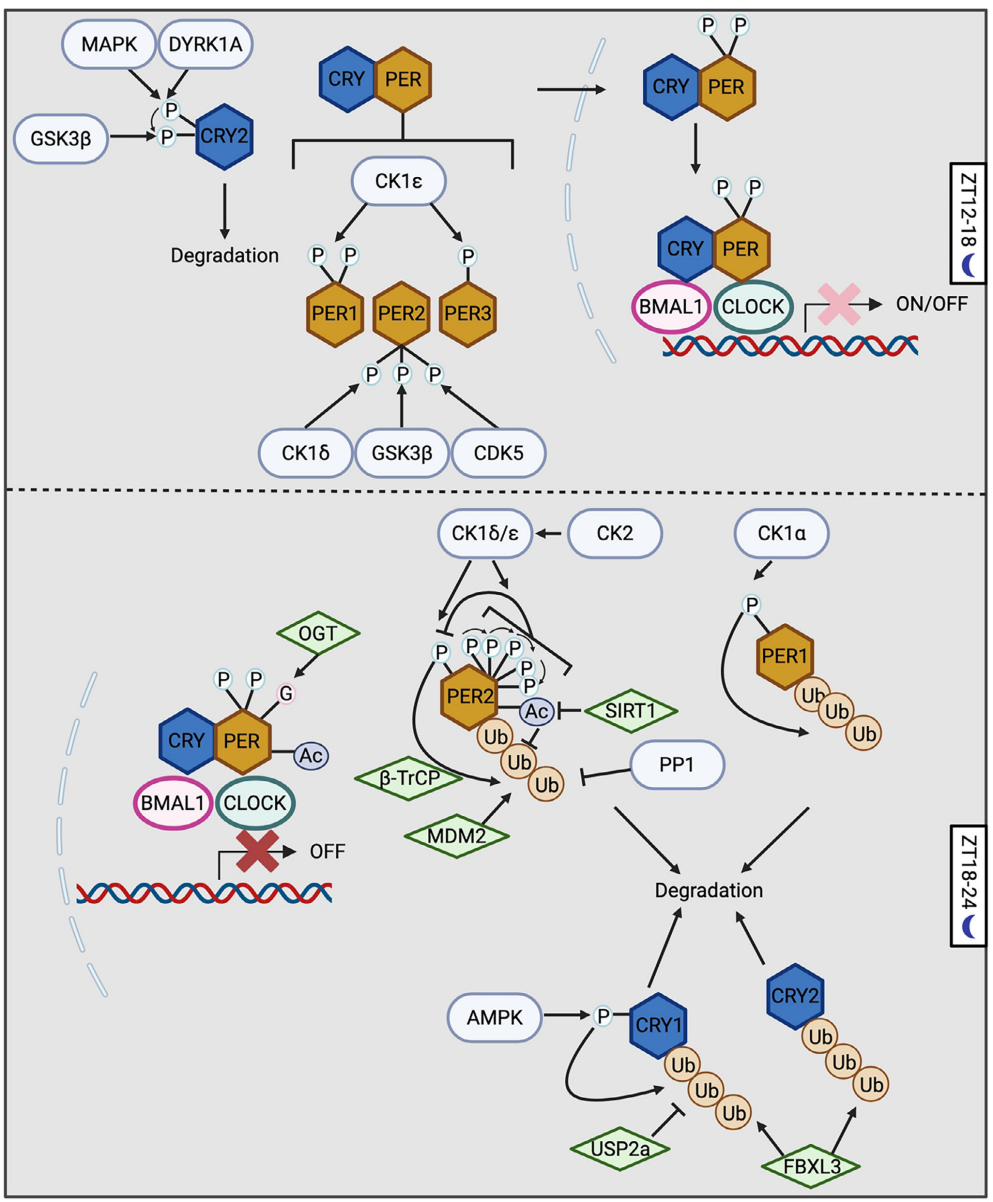
PTM–mediated regulation of negative elements in mammals. Prior to the repression phase of the circadian oscillator, early‐synthesized negative elements undergo degradation. MAPK and dual‐specificity tyrosine‐regulated kinase 1A (DYRK1A) phosphorylate CRY2, priming it for GSK3β‐mediated phosphorylation, which promotes CRY2 degradation. To facilitate the nuclear entry of negative elements during the first half of the night (ZT12–18), casein kinase 1ε (CK1ε) phosphorylates PER1 and PER3, whereas CK1δ, GSK3β and CDK5 phosphorylate PER2. During the repression phase, O‐GlcNAcylation of PER2 enhances its repressor activity. At the end of the repression phase, both PER and CRY proteins are degraded. PER2 degradation is controlled by a “phosphoswitch” mechanism, in which CK1δ/ε phosphorylates the FASP domain to inhibit subsequent phosphorylation at the β‐transducin repeat–containing protein (β‐TrCP) recognition site. β‐TrCP then binds to this site and mediates PER2 ubiquitination. CK2 further facilitates CK1δ/ε‐dependent PER2 degradation, while PP1 stabilizes PER2, presumably through dephosphorylation. Mouse double minute 2 homolog (MDM2) also targets PER2 for ubiquitination in a phosphorylation‐independent manner, whereas acetylation stabilizes PER2. For PER1, CK1α phosphorylates and destabilizes the protein. Both CRY1 and CRY2 are ubiquitinated by the F‐box and leucine‐rich repeat protein 3 (FBXL3). Additionally, AMP‐activated protein kinase (AMPK) phosphorylates CRY1 to promote its ubiquitination, while USP2a counteracts this process by deubiquitinating CRY1. Created with Biorender.com.

**FIGURE 5:**
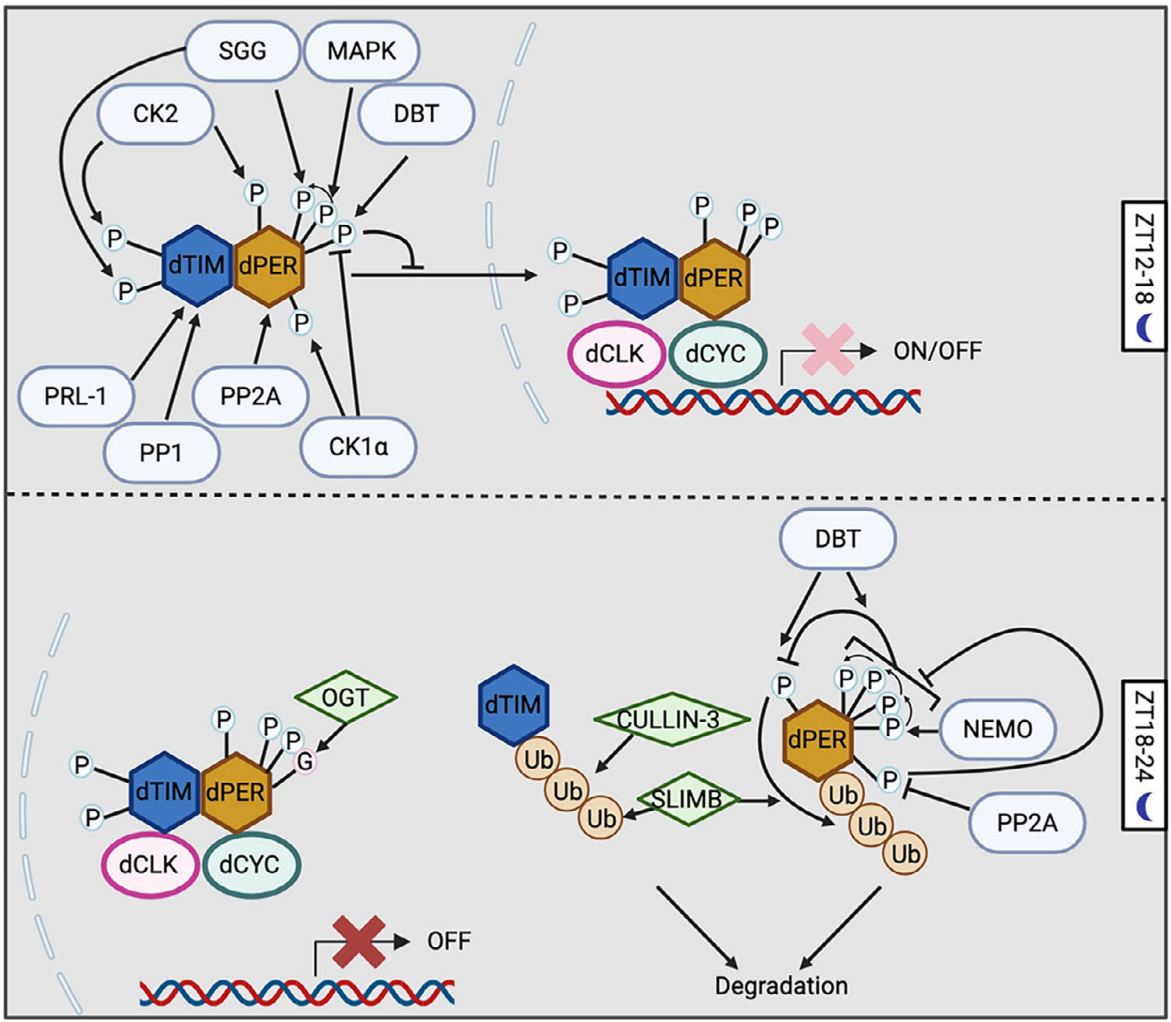
PTM‐mediated regulation of negative elements in *Drosophila*. DBT phosphorylates newly synthesized dPER to prevent its nuclear entry. CK2, CK1α, MAPK, Shaggy (SGG), and PP2A all promote dPER nuclear entry by modulating its phosphorylation status. dTIM facilitates the nuclear import of dPER through two mechanisms: 1) acting as a cargo protein that transports dPER into the nucleus; and 2) suppressing the inhibitory effect of DBT while enhancing the stimulatory function of CK2. Phosphorylation of dTIM by SGG triggers subsequent CK2‐directed phosphorylation of dTIM. In addition, phosphatase of regenerating liver‐1 (PRL‐1) and protein phosphatase 1 (PP1) increase the nuclear accumulation of dTIM. At the end of the repression phase, both dPER and dTIM are degraded. Supernumerary limbs (SLIMB) mediates the ubiquitination of dPER, whereas both SLIMB and CULLIN‐3 ubiquitinate dTIM. Similar to mammalian PER2, dPER is regulated by a “phosphoswitch” mechanism, although in this case two kinases—NEMO and DBT—are involved. Moreover, PP2A‐targeted sites can inhibit phosphorylation within the dPER short domain. Created with Biorender.com.

**FIGURE 6:**
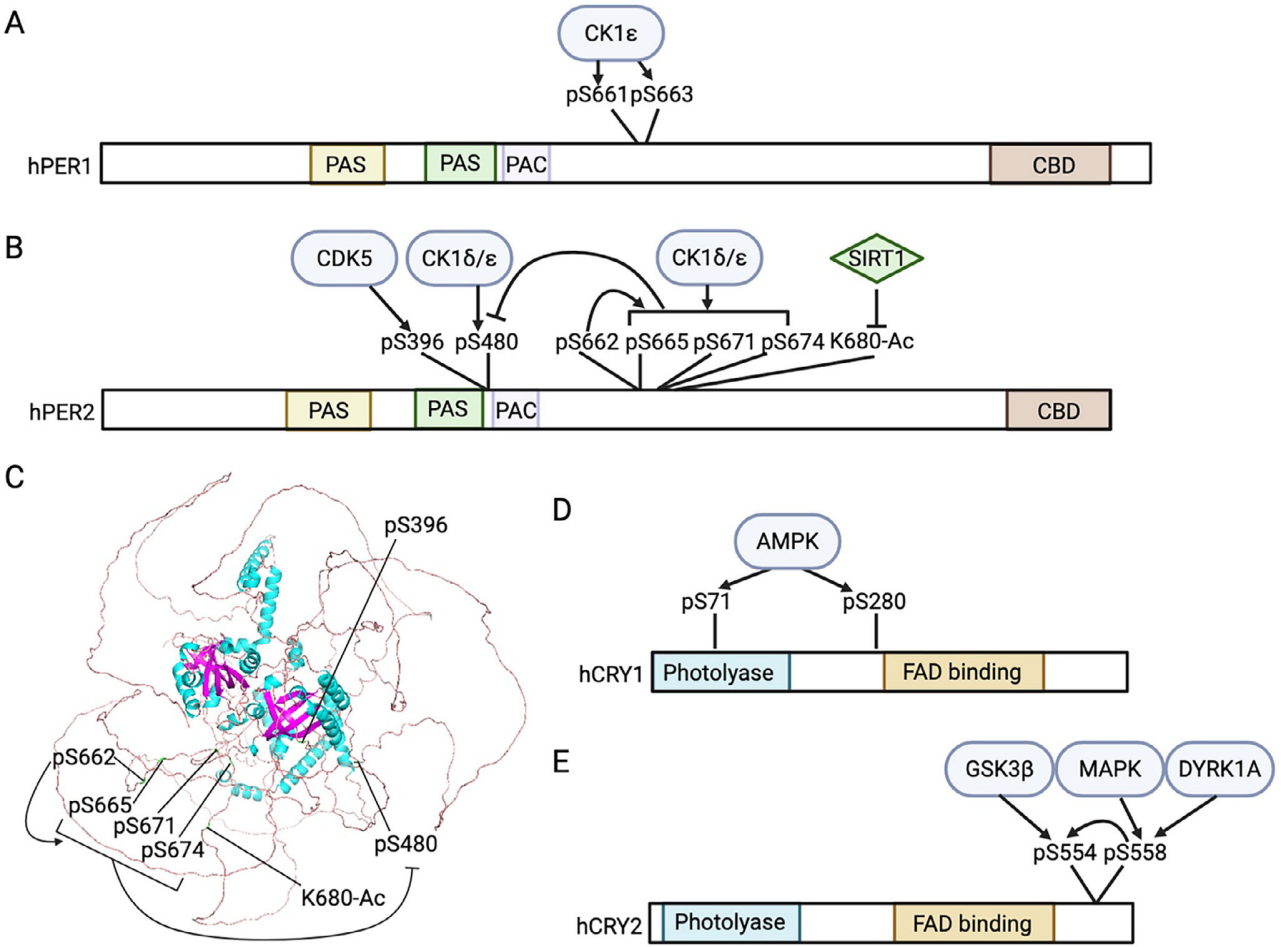
PTM landscape of negative elements in mammals. (A, B, D, E) Schematic representation of the functional modification sites on PER1 (A), PER2 (B), CRY1 (D), and CRY2 (E), as shown in Figure 4. (C) Structural model of PER2 highlighting the PTM sites in panel (B). Human PER1/2 and CRY1/2 are used to indicate the positions of modification sites, given the high sequence conservation of these proteins between humans and mice. Created with Biorender.com.

**FIGURE 7:**
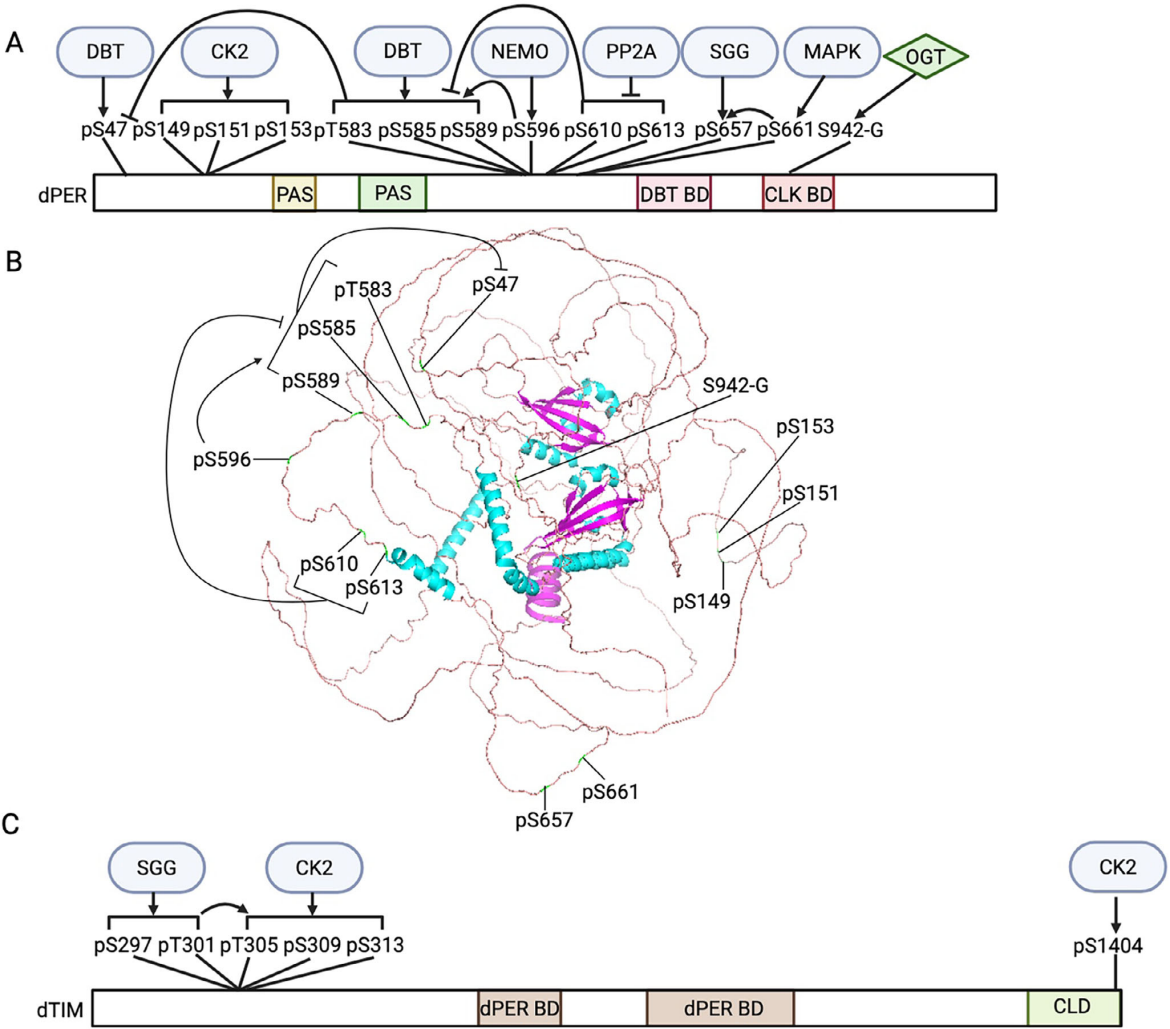
PTM landscape of negative elements in *Drosophila*. (A, C) Schematic representation of the functional modification sites on dPER (A), and dTIM (C), as shown in Figure 5. (B) Structural model of dPER highlighting the PTM sites in panel (A). Created with Biorender.com.

PTMs also regulate time‐of‐day specific function of negative elements, including nuclear entry, stability and repressor activity. As the positive elements of TTFL promote the expression of negative elements, the negative elements go through step‐wised PTMs, especially phosphorylation (Figures 4, 5, 6, 7, Table 1 and Table 2). In fact, among all circadian pacemaker proteins, dPER was first shown to be rhythmically phosphorylated [25]. Since 1998, DBT has been shown to phosphorylate dPER and different mutations of DBT can cause longer or shorter behavioral periods or arrhythmicity in flies [107, 108, 109, 110, 111, 112, 113, 114]. For mammalian clocks, the first circadian mutation discovered was the *tau* mutation of Syrian hamsters in 1988, which exhibits a significantly short behavioral rhythm as 20 h [115]. Twelve years later, the mutation was mapped to the gene coding for CK1ε homologue of DBT with a C to T transversion at residue 178 [116]. Later, extensive studies have shown that the casein kinases, CK1δ/ε in mammals and DBT in flies, are the main kinases in regulating the pace of molecular oscillator by targeting PER proteins, which is a conserved mechanism from flies to mammals [117]. In this section, we will summarize the PTM regulation of negative elements, highlighting how the step‐wised “phosphotimer” regulates the pace of molecular oscillator via controlling their degradation rate.

#### 2.2.1 Cellular Localization

Following translation, the negative elements must be imported into nucleus in order to exert their transcriptional repressor activity. This nuclear import process is temporally regulated, which creates the critical delay between the expression of negative elements and the onset of feedback repression. In the mammalian clock, while the interaction of PER1/2 proteins with CRYs or PER3 is important to regulate their subcellular localization, nuclear localization of PER1/2 in the case of lacking both CRYs or PER3 is also observed [31, 32, 118], indicating additional regulatory mechanisms. Phosphorylation poises another layer of regulation (Figure 4 and Figure 6, Table 1). CK1ε was shown to phosphorylate all three PERs proteins, and increase the nuclear localization mPER1 and mPER3 but not mPER2 [119]. Although the effect of CK1ε on mPER3 nuclear entry was confirmed in later studies [120, 121], in 2000, another study suggested the opposite function of CK1ε that CK1ε‐directed phosphorylation of mPER1 masks mPER1 NLS (aa824‐851) and leads to cytoplasmic retention [122]. To solve this controversy and investigate the precise molecular mechanism of CK1ε‐regulated mPER1 nuclear localization, Takano et al. [123] identified three CK1ε‐directed phospho‐clusters in mPER1 and found that phosphorylation of mPER1 S661 and S663 is important for the nuclear entry of mPER1. This highlights the importance of investigating the function of individual PTM sites for PTM studies, instead of relying on the modulation of the overall activity of modifiers. As for PER2, the nuclear localization is mainly regulated by CK1δ [124] and GSK3β [125]. Pharmaceutical inhibition of both CK1δ and GSK3β causes longer period at behavioral and molecular level [125, 126, 127]. Iitaka et al. [125] showed that GSK3β phosphorylates rPER2 and promotes nuclear entry of rPER2. In addition, CDK5 phosphorylates mPER2 at S394 to promote nuclear entry [128, 129]. However, site‐specific studies are necessary to reveal the mechanism by which PER proteins enter the nucleus, and whether the nuclear localization of CRY proteins, the partners of PER, is regulated by PTMs remains unknown.

In flies, the nuclear localization of dPER/dTIM complex is dynamically regulated by phosphorylation (Figure 5 and Figure 7, Table 2). In the cytoplasm, newly synthesized dPER is phosphorylated by DBT, which inhibits nuclear entry [130, 131]. However, CK2 [132, 133, 134, 135], CK1α [136], MAPK [137, 138], and Shaggy (SGG, the *Drosophila* homologe of GSK3β) [138] promotes dPER nuclear entry, which is evident that disturbing the activity of these kinases decreases nuclear dPER and results in longer behavioral period. The dominant negative CK2α^tik^ mutant contains two site mutations, including M161K and E165D [132]. Expressing CK2α^tik^ in clock neurons results in up to 33 h locomotor period [133]. CK2α is shown to phosphorylate dPER at S149, S151 and S153 [135], which promotes the nuclear entry of dPER [132, 133, 135]. Not surprisingly, the *Andante* mutant, mutation of CK2β which decreases CK2 α2:β2 holoenzyme level, also exhibits longer behavioral period and delayed dPER nuclear entry [134], indicating that CK2 regulates dPER as a holoenzyme. Functional characterization of CK1α in molecular oscillators showed that CK1α‐dependent dPER phosphorylation promotes nuclear localization by inhibiting DBT‐dependent phosphorylation in cytoplasm [136]. MAPK phosphorylates dPER at S661, which primes the SGG‐direct phosphorylation of dPER S657 and triggers nuclear entry of dPER [138]. Moreover, phosphatases also affect the nuclear entry of dPER. Overexpression of *widerborst* (*wdb*), a subunit of PP2A, in clock neurons results in advanced dPER nuclear accumulation [139].

One of the functions of dTIM in the circadian clock is to promote the nuclear entry of dPER [140, 141, 142]. dTIM can bind to importin α1 and act as a cargo protein to transport dPER into nucleus.[143] On the other hand, dTIM also impacts the mechanisms described above on dPER nuclear entry (Figure 5 and Figure 7, Table 2). dTIM can counteract the inhibitory effect of DBT on dPER nuclear entry [130]. dTIM is also required for CK2 to control dPER subcellular localization [144]. SGG is also involved in the impact of CK2 on dPER/dTIM localization. Indeed, overexpression of SGG leads to shorter behavior period due to the premature nuclear entry of dPER/dTIM [145]. Kinase assay in vitro showed that SGG phosphorylates dTIM at S297 and/or T301, which stimulates the phosphorylation of dTIM T305, S309 and S313 by CK2 [146]. These SGG‐ and CK2‐directed phosphosites increase nuclear accumulation of dPER/dTIM in the pacemaker neurons, sLNvs [146]. Other than kinases, two phosphatases, phosphatase of regenerating liver‐1 (PRL‐1)[147] and phosphatase 1 (PP1) [148], was also found to increase the nuclear level of dTIM. PRL‐1 was found to reduce the phosphorylation of S586, a site adjacent to nuclear localization signal of dTIM [147]. However, it remains to be investigated which kinase(s) the functions of these phosphatases counterpart.

The nuclear export of negative elements is important for preventing pre‐mature inhibition of positive elements. In mammals, PER2 nuclear export is slower compared to import [33], and exported PER2 is rapidly ubiquitinated and degraded [149]. For CRY1 and CRY2, PER1 can mediate their nuclear export [34]. In flies, dPER is likely essential for the nuclear retention of dTIM [150]. On the other hand, by identifying the phosphosites on dPER‐bound dTIM, Cai et al. [30] demonstrated that CK2‐dependent dTIM S1404 phosphorylation increases the nuclear accumulation of dTIM and dPER by inhibiting the interaction between dTIM and exportin 1.

In conclusion, the nuclear entry of negative elements requires the formation of repressor complexes, and CK1 and GSK3β/SGG are the main kinases that stimulate their nuclear entry in both *Drosophila* and mammals (Figures 4, 5, 6, 7, Table 1 and Table 2). However, the precise mechanisms by which individual phosphosites dictate nuclear import versus retention are not fully resolved, and how kinase‐phosphatase networks are coordinated across tissues and integrated with environmental cues remains poorly understood.

#### 2.2.2 Degradation to Regulate the Speed of Molecular Oscillator

After the inhibition of the positive elements, negative elements degrade and the subsequent cycle starts. The degradation rate of negative elements, especially PER proteins, is precisely controlled by PTMs, which is cardinal for the maintenance of 24 h period of the molecular oscillator (Figures 4, 5, 6, 7, Table 1 and Table 2). Mechanisms by which PERs degrade show notable conservation in flies and mammals: 1) CK1 is the main regulatory kinases of PER stability; 2) depending on the phosphorylated residues, some stabilize PERs while others trigger PER degradation through proteasomal pathway 3) β‐transducin repeat containing protein 1 and 2 (β‐TrCP1 and 2) and Supernumerary limbs (SLIMB, the fly homologue of β‐TrCPs) are the E3 ubiquitin ligases that modify PER proteins (Figures 4, 5, 6, 7, Table 1 and Table 2). These mechanisms were first discovered in flies [35, 36], and later Virshup group summarized and named it “phosphoswitch” [37].

In flies, early studies isolated several DBT mutants, and found that different DBT mutations lead to various behavioral phenotype, including long, short periods and arrhythmicity [107, 108, 110, 111, 131, 151]. Many studies found that these DBT mutants alter the phosphorylation status and level of dPER protein [107, 108, 110, 111, 131]. These phenomena could be simply explained by different enzymatic activity level of DBT. However, later studies suggested that both DBT^S^ and DBT^L^ variants show lower kinase activity, which were not in line with the period shortening and lengthening phenotype of flies bearing *dbt^S^* and *dbt^L^* alleles, respectively [112]. This suggests that the regulation of dPER by DBT is rather complicated. In an analysis of *dbt^ar^* flies, an arrythmic mutant, Rothenfluh et al. [113] found that the previously discovered *per^s^* mutant, which contains a S589N mutation with a ∼19 h period [152], can restore the rhythms in behavior and molecular oscillator of *dbt^ar^*. These observations indicates that DBT may regulate dPER through S589 and the surrounded the “per‐short domain” (aa585‐601), mutation of residues in which mostly results in short period [152, 153]. Indeed, mass spectrometry (MS) analysis showed that DBT phosphorylates sites around the dPER short domain, including S589 [35, 114].

In addition to the per‐short domain, DBT also phosphorylates dPER at multiple phopho‐clusters [35]. Since the hyperphosphorylated dPER as a result from progressive phosphorylation by DBT is rendered for degradation, Ko et al. [154] and Grima et al. [155] investigated the role of phosphorylation‐primed ubiquitin ligases and found that SLIMB, a F‐box protein of the SCF complex, mediates the proteasomal degradation of dPER. Later, in an effort to identify the regions regulating dPER degradation, Chiu et al. [35] found that dPER S47 phosphorylation is required for SLIMB‐dPER interaction, while DBT phosphorylates dPER S47 in vitro and in S2 cells. Subsequently, Chiu et al. [36] investigated the role of per‐short domain in regulating dPER stability and its association with PER S47. They found that NEMO phosphorylates dPER at residue S596, which triggers the DBT‐mediated phosphorylation of residues T583, S585 and S589. This phosphor‐cluster, in turn inhibits S47 phosphorylation to stabilize dPER [36]. This regulatory model is supported by an independent work from Hardin group, which demonstrated that NEMO could slow down the pace of circadian clock [156]. In vivo study of the PP2A targeted phosphosites further identified T610 and S613 in the “Per‐Short Downstream (Per‐SD)” domain, which inhibit the phosphorylation of per‐short domain to promote dPER degradation [157].

In mammals, progressive phosphorylation has also been shown to modulate PER2 degradation (Figure 4 and Figure 6, Table 1). Extensive studies, including pharmacological inhibitors or activators in cells [121, 158, 159], mutations or overexpression of CK1δ/ε in cells [124, 160, 161] and mice [162], and mutation of PER2 sites for CK1δ binding [163], all support that CK1δ/ε regulate the stability of PER2 proteins. Much like per‐short domain in flies, the mammalian PER2 contains a familial advanced sleep phase (FASP) domain [164]. Patients with FASP syndrome exhibit a 4 h advanced sleep phase due to mutation that causes amino acid substitution at hPER2 S662 [165]. This FASPS mutation (S659 in mPER2) has been shown to reduce mPER2 stability by increasing CK1ε‐mediated degradation [166]. Similar to the phosphorylation cascade in per‐short domain in flies, phosphorylation of hPER2 S662 primes the phosphorylation of S665, S668, S671 and S674 by CK1δ/ε [167, 168]. Phosphorylation of this FASP domain enhances the stability of PER2 [169]. It has also been shown that CK1δ/ε phosphorylate the priming site mPER2 S659 as well, with the CK1δ2 splice variant and CK1ε being more active than CK1δ1 [168, 170], revealing additional layer modulating PER2 phosphorylation.

Regarding the phosphodegron and E3 ubiquitin ligases of mPER2, β‐TrCP1 and 2, the homologs of fly SLIMB, have been found to mediate mPER2 degradation by recognizing the phosphorylation events catalyzed by CK1δ/ε [171, 172, 173, 174]. Tissue culture and mouse experiments have demonstrated that mPER2 S478 is the key CK1δ/ε phosphosite that serve as the phosphodegron recoganized by β‐TrCPs [37, 171, 173, 175]. By monitoring the kinetics of PER2::LUC degradation, Zhou et al. [37] observed a three‐stage process for PER2 degradation. Mathematical modeling further predicted a “phosphoswitch”: phosphorylation of the FASP domain inhibits the phosphorylation of the phosphodegron site to stabilize PER2, while phosphorylation of phosphodegron site targets PER2 for rapid degradation [37]. Finally, the Partch group performed NMR kinase assays and found that the phosphorylation of FASP domain is rate‐limited by the phosphorylation of S662 priming site [176]. The phosphorylation of the FASP domain inhibits the phosphorylation of phosphodegron site by binding and inhibiting CK1 kinase activity [176]. With the parallel analysis on dPER, they demonstrated a conserved mechanism by which phosphorylation at per‐short domain also inhibits DBT‐dependent phosphorylation at S47 [176].

The phosphoswitch model is important as it provides interpretation for several observations. First, it explains how reduced CK1/DBT kinase activity enhances PER degradation by stimulating PER phosphorylation [177, 178, 179, 180]. The CK1ε^tau^ mutant exhibits reduced activity toward the primed FASP domain, but retains activity at the non‐priming dependent phosphodegron site [37], which is further supported by recent structural study of CK1 [181]. Second, mathematical modeling highlights this bistable phosphoswitch enables synchronized nuclear entry of PER molecules under noisy cellular conditions, such as varying cytoplasmic congestion levels, cell sizes and activator levels [182, 183]. It is predicted that perinucleus PER accumulates to a threshold level to trigger the “phosphoswitch”, which results in synchronized phosphorylation, nuclear entry and timely repression [182]. Finally and interestingly, the “phosphoswitch” model also provides molecular explanation for the temperature compensation of the circadian clock, which will be discussed in section 3.3.

Beyond DBT and CK1δ/ε, PER stability is also regulated by other kinases, phosphatases, and additional PTMs in both flies and mammals (Figures 4, 5, 6, 7, Table 1 and Table 2). In flies, ribosomal S6 kinase (S6K) [184, 185], and PP2A [139, 157] stabilize dPER, whereas CK1α promotes DBT‐dependent PER phosphorylation and degradation in nucleus [136]. In mammals, PP1 inhibits mPER2 degradation [177]; CK2 enhances CK1ε‐dependent PER2 degradation [186], while its direct phosphorylation of PER2 stabilize PER2 [187]; and CK1α phosphorylates an destabilizes PER1 [188]. In addition to phosphorylation, O‐GlcNAcylation has been found to stabilize dPER in flies [189]. In mammals, SIRT1 deacetylates PER2 at lysine 680 (K680), reducing PER2 stability [190, 191]. Furthermore, mouse double minute 2 homolog (MDM2)‐a ubiquitin ligase‐targets PER2 for ubiquitination and degradation independent of phosphorylation [192]. However, the specific amino residues targeted by these kinases, phosphatases, OGT, and MDM2 to regulate PER stability still remain to be investigated.

In addition to PER, the stability of other proteins of the repressor complex is also regulated by phosphorylation and ubiquitination (Figure 4 and Figure 6, Table 1). In mammals, three research groups simultaneously identified FBXL3, the ubiquitin ligase of CRY1 and CRY2, through genetic screen in 2007 [38, 39, 193]. Two mutations identified, *Afterhours* (*Afh*, FBXL3 C358S) and *Overtime* (*Ovtm*, FBXL3 I364T), both lead to longer behavioral period, due to slower degradation of CRY1 and CRY2 [38, 193]. Crystallographic studies revealed that FBXL3 promotes degradation of mCRY1 and mCRY2 by interacting with their FAD‐binding pocket and masking its mPER‐binding interfaces [194, 195], indicating that both FAD and mPERs can interfere with FBXL3‐dependent mCRY1/2 degradation. Conversely, USP2a has been found to stabilize CRY1 through deubiquitination [196]. As for phosphorylation, AMPK regulates the expression of clock genes and the pace of circadian clock [197, 198]. Mechanistic studies demonstrated that AMPK phosphorylates mCRY1 at S71 and S280, enhancing CRY1 degradation via facilitating mCRY1‐FBXL3 binding [199]. Importantly, the nuclear localization AMPKα1 (the catalytic subunit of AMPK) peaks at subjective day time in mouse liver, coinciding with the peak interaction between mCRY1 and FBXL3 and lower nuclear mCRY1 level, indicating that AMPK regulates the rhythmic abundance of mCRY1 [199, 200]. Besides, GSK3β phosphorylates mCRY2 at S553, triggering its degradation [201, 202]. Two kinases are found to phosphorylate mCRY2 S557 that primes S553 phosphorylation by GSK3β, including MAPK [203] and DYRK1A [202]. Both S557 phosphorylation and GSK3β activity is rhythmic and peak at late night to early morning in mouse liver, overlapping with the accumulation phase of mCRY2 [201, 202]. However, FBXL3 is shown to be irrelevant to the pS553/pS557 regulated mCRY2 degradation, suggesting the involvement of other E3 ubiquitin ligase [202]. Based on the subcellular localization of the kinases and FBXL3, pS553/pS557 mainly regulate the degradation of cytosolic mCRY2 and therefore delaying nuclear entry, while FBXL3 clears the nuclear mCRY2 at the end of repression phase [38, 202]. In flies, the stability of dTIM, the functional partner of dPER, is tightly regulated by environmental light signals, which will be discussed in section 3.1.

In summary, the temporal stability of PER proteins is governed by a conserved phosphorylation‐dependent “phosphoswitch” mechanism in both *Drosophila* and mammals (Figures 4, 5, 6, 7, Table 1 and Table 2). In this system, CK1 homologs‐DBT in flies and CK1δ/ε in mammals‐phosphorylate PER at multiple, functionally distinct clusters/residues that modulate degradation. This dual‐site regulatory logic underpins the robustness to achieve ∼24 h periodicity of the circadian molecular oscillator. Moving forward, a major challenge will be to comprehensively map the full spectrum of post‐translational modifications on PER and CRY proteins, to elucidate the dynamic interplay among phosphorylation, ubiquitination, O‐GlcNAcylation, and acetylation across temporal and cellular contexts.

#### 2.2.3 Regulation of Repressor Activity

In many cases, the repressor function of negative elements is regulated by modulation of protein stability and/or nuclear localization [30, 133, 138, 159, 184, 204, 205] (Figures 4, 5, 6, 7, Table 1 and Table 2). For example, AMPK activation stimulates the phosphorylation and activity of CK1ε, which results in faster mPER2 degradation rate, reduced repression and advanced *mPer1/2* mRNA rhythms in heart and muscle tissues [159]. Restoring nuclear localization of *dperΔ*, a mutation of dPER‐DBT binding domain, rescues the reduced repressor activity of this mutant [205].

However, in other cases, the same site can simultaneously regulate PER stability and repressor activity (Figures 4, 5, 6, 7, Table 1 and Table 2). The FASPS‐associated variant, hPER2 (S662G), is a more potent repressor than wild‐type hPER2 [167]. Interestingly, O‐GlcNAcylation also occurs at S662 that enhances repressor activity, demonstrating interplay of PTMs on the regulation of circadian clocks [206]. In flies, by contrast, O‐GlcNAcylation of dPER at S942 inhibits dPER repressor activity by preventing premature dPER‐dCLK interaction [207]. SIRT1‐dependent acetylation not only reduces mPER2 stability but also modulates PER2/CRY1 binding and therefore repressor activity [191]. In flies, dPER S589 phosphorylation increases its repressor activity [114, 208].

Moreover, there is evidence that CK1δ/ε regulate PER2 repressor activity. Luciferase reporter assay showed that CK1δ/ε reduce the repressor activity of mPER2, while enhance that of mPER3 [120]. More recently, mutation of the sites mediating PER2 and CK1δ interaction in mice revealed that this interaction plays two opposite roles in the molecular oscillator: 1) reducing the activity of BMAL1/CLOCK by inhibiting their E box binding; 2) suppressing the repressor function of CRY1 by decreasing CRY1 occupancy at E box [163]. Finally, comprehensive characterization of mCRY1 phosphosites identified that the phospho‐cluster (aa243‐258) is important for period determination, independent of mCRY1 stability [209].

In summary, it is intriguing to find that the same PTM site and kinase modulate the function of negative elements via multiple aspects in both flies and mammals (Figures 4, 5, 6, 7, Table 1 and Table 2). Future studies should aim to dissect the physiological significance of PTMs interplay on the same or nearby residues to fine‐tune repressor function, elucidate structural insights revealing modification patterns to transcriptional inhibition, and determine how these regulatory networks adapt across tissues and pathological conditions to maintain the pace of the circadian clock.

## 3 PTMs in Response to Environmental Factors

### 3.1 Light

**FIGURE 8:**
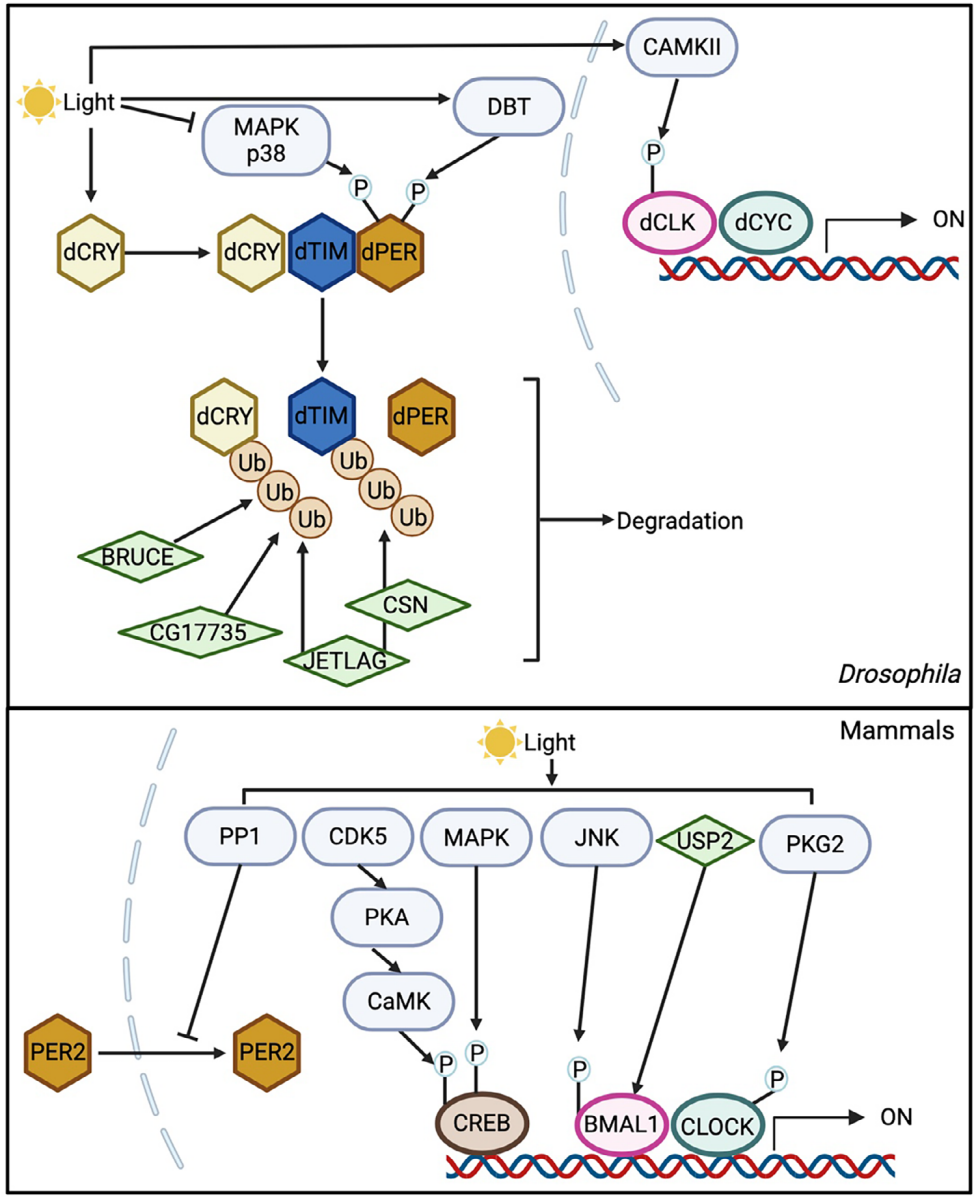
Regulation of circadian clocks by light through PTM mechanisms. In *Drosophila*, light resets the molecular oscillator primarily by promoting dTIM degradation. Light activates dCRY, which triggers COP9 signalosome (CSN)‐mediated dTIM degradation through the ubiquitin ligase JETLAG. Meanwhile, dCRY itself is ubiquitinated by JETLAG, BRUCE, and CG17735 and subsequently targeted for degradation. In addition, light regulates dCLK modification via calcium/calmodulin‐dependent protein kinase II (CaMKII) and modulates dPER phosphorylation through DBT and MAPK p38. In mammals, light resets the circadian oscillator by stimulating the transcription of core clock genes. This process is mediated by CREB phosphorylation through inhibition of PER2 nuclear entry by PP1, CREB phosphorylation by CDK5‐PKA‐CaMK cascade and MAPK signaling, BMAL1 phosphorylation by c‐Jun N‐terminal kinase (JNK), BMAL1 deubiquitination by USP2, and CLOCK phosphorylation by protein kinase G2 (PKG2). Created with Biorender.com.

Light is the primary zeitgeber for the circadian timing system. In mammals, the master clock in SCN receive light signals through intrinsically photosensitive retinal ganglion cells (ipRGCs) in the visual system and retinohypothalamic tract (RHT), and light‐induced resetting of the molecular oscillators is mediated by acute expression of clock genes (e.g., *Per1*) [12] (Figure 8). In *Drosophila*, the blue light photoreceptor, dCRY, in clock neurons can directly receive light signals and trigger the degradation of dTIM (and subsequent destabilization of dPER protein) [210] (Figure 8, Table 2). Throughout these processes, PTMs serve as key regulatory mechanisms modulating the activity of relevant proteins, as detailed below.

In the 1990s, studies demonstrate that light reduces the level of dPER proteins by disruption dPER‐dTIM interaction in *Drosophila* [211, 212, 213, 214]. Sehgal group found that light induces dTIM degradation through tyrosine phosphorylation and ubiquitination [215, 216]. As for the upstream photic responsive pathway, the visual system is dispensable for the light entrainment of flies, which is different from mammals [217, 218, 219]. Instead, dCRY has been identified as the dedicated cellular photoreceptor for the resetting of circadian rhythms [220, 221, 222]. During the dark phase, dCRY level is high and inhibits the ubiquitination of dTIM[223]. In the light phase, dCRY undergoes conformational changes and promotes dTIM for proteasomal degradation. Inhibition of dCRY's electron transport enhances dTIM ubiquitination, indicating that light‐induced conformational changes of dCRY contribute to dTIM ubiquitination [223].

Wild‐type flies become arrhythmic under constant light (LL) condition due to dTIM degradation. Therefore, fly mutants exhibiting behavioral rhythmicity under LL provide opportunities investigating molecular mechanisms by which light resets circadian clocks. Sehgal and Stanewsky groups identified JETLAG, an F‐box E3 ubiquitin ligase, that regulates the light‐dependent dTIM degradation [224, 225]. Additionally, the COP9 signalosome (CSN), a regulator of SCF‐type E3 ligases, mediates the effect of JETLAG on dTIM [226]. Later, JETLAG was also shown to mediate light‐induced degradation of dCRY in S2 cells [227, 228]. Further RNAi screening in S2 cells identified over 190 potential candidates involved in mediating the light‐induced dCRY degradation. Of these candidates, 3 were validated in flies, including two ubiquitin ligases, *Bruce* and *CG17735* [229, 230]. Moreover, the only known kinase that regulates the light sensitivity of dTIM and dCRY is SGG [231, 232]. However, these two studies led to opposite conclusions: Yuan et al. [232] showed that light‐induced serotonin signaling inhibits SGG activity and stabilizes dTIM, whereas Stoleru et al. [231] found that overexpression of SGG in clock neurons primarily stabilizes dCRY, which in turn increases dTIM stability. This discrepancy might arise due to that modulating serotonin signaling induces additional changes in clock neurons in addition to SGG activity, which requires further investigations. Phosphatases also regulate light sensitivity of dTIM. Different light conditions alter the period length of *PRL‐1* mutants; as mentioned earlier (section 2.2.1), PRL‐1 dephosphorylates dTIM and regulates its nuclear entry [147]. Finally, it is worth noting that, beyond light‐induced dTIM degradation, dTIM protein also undergoes degradation and exhibits oscillations under constant darkness condition for circadian timekeeping in absence of light. This process is mediated by the E3 ligases SLIMB and CULLIN‐3 [155, 233, 234].

Other than dTIM and dCRY, light also modulate the PTMs of other clock proteins in *Drosophila* (Figure 8, Table 2). For instance, calcium signaling and Calcium/calmodulin‐dependent protein kinase II (CaMKII)‐directed phosphorylation of dCLK may mediate light induced response [235]. Light can also induce dPER at residues S826/S828 by DBT, which affects behavioral rhythms without altering the stability, localization and repressor activity of dPER [236]. Additionally, DBT might be involved in light‐induced responses through its regulators, including *spaghetti* and *bride of doubletime* [237, 238, 239]. Importantly, the MAP kinase p38 may represent a conserved mechanism for light entrainment across mammals [240, 241] and flies [137]. In flies, p38 activity is inhibited by light and p38 mutation delays the nuclear entry of PER [137]. In vitro kinase assay further showed that p38 phosphorylates dPER at residues S661 and S975 [137].

Since light resets the circadian clock by triggering the expression of clock genes in mammals, such as *Per1*, it is plausible that light also regulates the function of BMAL1/CLOCK activator complex and the relevant chromatin landscape. In 2010s, light has been shown to modulate BMAL1 through the c‐Jun N‐terminal kinase (JNK) [242] and the deubiquitinase USP2 [64]. Addtionally, PKG2 phosphorylates CLOCK in response to light pulse at night [243]. Light pulse at night also increases the phosphorylation of Histone H3 at S10 in SCN, which correlates with the expression of *Per1* and is inhibited by GABA signaling [244]. Light could also promote gene expression by inhibiting the function of negative elements. The reduction of PP1 function enhances the phase shift during light‐induced clock resetting by increasing the nuclear accumulation of PER2 [245]. Furthermore, *Per1* promoter also contains four cAMP response elements and is regulated by cAMP response element‐binding protein (CREB) [246]. Light pulse during night activates CREB via CaMK and MAPK signaling pathways, thereby inducing *Per1* expression [240, 241]. CDK5 can also trigger the PKA‐CaMK‐CREB cascade during light entrainment of SCN[247]. Consistent with the observations in flies, the main clock kinase CK1ε also regulates light entrainment, as CK1ε^−/−^ mouse or CK1ε inhibitor enhance phase resetting of behavioral rhythm in response to light pulses and shifts in light/dark cycles [248, 249]. However, the detailed mechanisms by which CK1ε mediates light response remain unknown.

Taken together, light resets the circadian clock through distinct pathways in flies and mammals: in flies, light‐induced dCRY activation promotes the ubiquitin‐dependent degradation of dTIM‐via JETLAG; in mammals, photic signals drive *Per1* transcription through MAPK‐, CaMK‐, and CDK5‐mediated pathways modulating CREB, BMAL1, CLOCK, and histones (Figure 8). The involvement of kinases such as MAPK (p38 in *Drosophila*) and CK1ε (DBT in *Drosophila*) in both organisms suggests the evolutionary conservation of kinase‐based light entrainment mechanisms (Figure 8, Table 1 and Table 2). Given the CK1 homologs are involved in light entrainment and temperature compensation (discussed in section 3.3), it would be interesting to determine how light‐induced modifications interface with temperature compensation, and environmental adaptability of the circadian system in the future.

### 3.2 Nutrition and Metabolism

Nutrient availability or feeding‐fasting cycles is another important zeitgeber for peripheral clocks. In tissues, such as liver, the molecular oscillator and the rhythmic transcriptome is largely regulated by feeding activity [15, 250, 251, 252, 253]. From the perspective of PTMs, it is highly possible that the nutrient‐responsive kinases mentioned earlier mediate the impact of metabolism on the molecular oscillator in flies and mammals, including GSK3β/SGG [61, 62, 125, 126, 130, 145, 146, 201, 254, 255, 256, 257], and AMPK [76, 159, 197, 198, 199]. Zhang et al. [258] specifically investigated the mechanisms by which time restricted feeding (TRF) entrains circadian clocks in cerebral cortex. They found that PKCγ mediates the TRF‐induced phase shift of clock gene expression by reducing ubiquitination and thus stabilization of BMAL1. Beyond phosphorylation, O‐GlcNAcylation, acetylation and S‐palmitoylation of clock proteins also link the molecular oscillator to metabolism. O‐GlcNAcylation is nutrient sensitive because it relies on UDP‐GlcNAc as substrate that is produced from hexosamine biosynthetic pathway (HBP), which integrates metabolites from glucose, amino acid, lipid and nucleotide metabolism [77, 78]. As mentioned above, O‐GlcNAcylation modifies and regulates dPER [189, 207], PER2 [206], CLOCK [69], and BMAL1 [69]. SIRT1 requires metabolically‐sensitive NAD^+^ as cofactor to deacetylates BMAL1 [102, 103], and PER2 [190]. The substrate of S‐palmitoylation is metabolized from acetyl‐CoA and the most common fatty acid in animals, palmitic acid [68]. Thus, the S‐palmitoylation of CLOCK may interfaces lipid metabolism and the molecular clock [68].

Importantly, key nutrient sensors, including GSK3β/SGG [61, 201], AMPK [199], O‐GlcNAcylation [259, 260], and SIRT1 [102], exhibit daily rhythmicity. Particularly, O‐GlcNAcylation and NAD^+^/SIRT1 interplay with the circadian clock. In flies, the rhythmic enzymatic activity of GFAT (the rate‐limiting enzyme of HBP) is governed by the molecular clock [259]. In mice, the rhythmic expression of nicotinamide phosphoribosyltransferase (NAMPT), the rate‐limiting enzyme of NAD^+^ salvage pathway, is driven by BMAL1/CLOCK [104, 261]. Since daily rhythms have been observed in acetyl‐CoA and *Acsl1* mRNA, the gene encoding enzymes for generating substrate for S‐palmitoylation [68], S‐palmitoylation may also oscillate. Moreover, these nutrient sensors crosstalk, creating intricate regulatory networks [78, 198]. For example, AMPK mutant mice exhibits disrupted *Nampt* mRNA rhythm and reduced NAD^+^ level [198], demonstrating the regulation of NAD^+^/SIRT1 pathway by AMPK.

**FIGURE 9:**
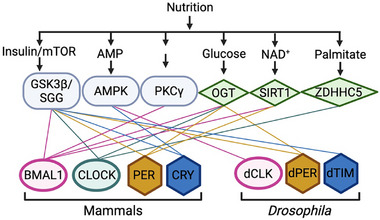
Nutritional regulation of clock proteins via PTMs. Nutrition regulates the PTMs of mammalian and *Drosophila* clock proteins through nutrient‐sensitive pathways and metabolic sensors, including GSK3β/SGG, AMPK, PKCγ, OGT, SIRT1, and ZDHHC5. Created with Biorender.com.

In both *Drosophila* and mammals, kinases such as GSK3β/SGG and AMPK regulate core clock proteins in response to metabolic cues, while additional PTMs‐including O‐GlcNAcylation, acetylation, and S‐palmitoylation‐also modulate the metabolic response of the circadian clock (Figure 9, Table 1 and Table 2). Notably, O‐GlcNAcylation and the NAD^+^/SIRT1 axis exhibit rhythmicity and are themselves under circadian control, which reflects a bidirectional regulation between metabolism and the clock. Importantly, with the technical improvement of mass spectrometry, accumulating evidence has shown that these nutrient‐sensitive PTMs can rhythmically modify a repertoire of proteins beyond the molecular oscillator, thereby achieving temporal regulation of proteome [260, 262, 263, 264]. Finally, it will be intriguing to investigate the physiological relevance of nutrient‐sensitive PTMs in the interface of circadian rhythms and metabolic homeostasis, aging, and disease.

### 3.3 Temperature

**FIGURE 10:**
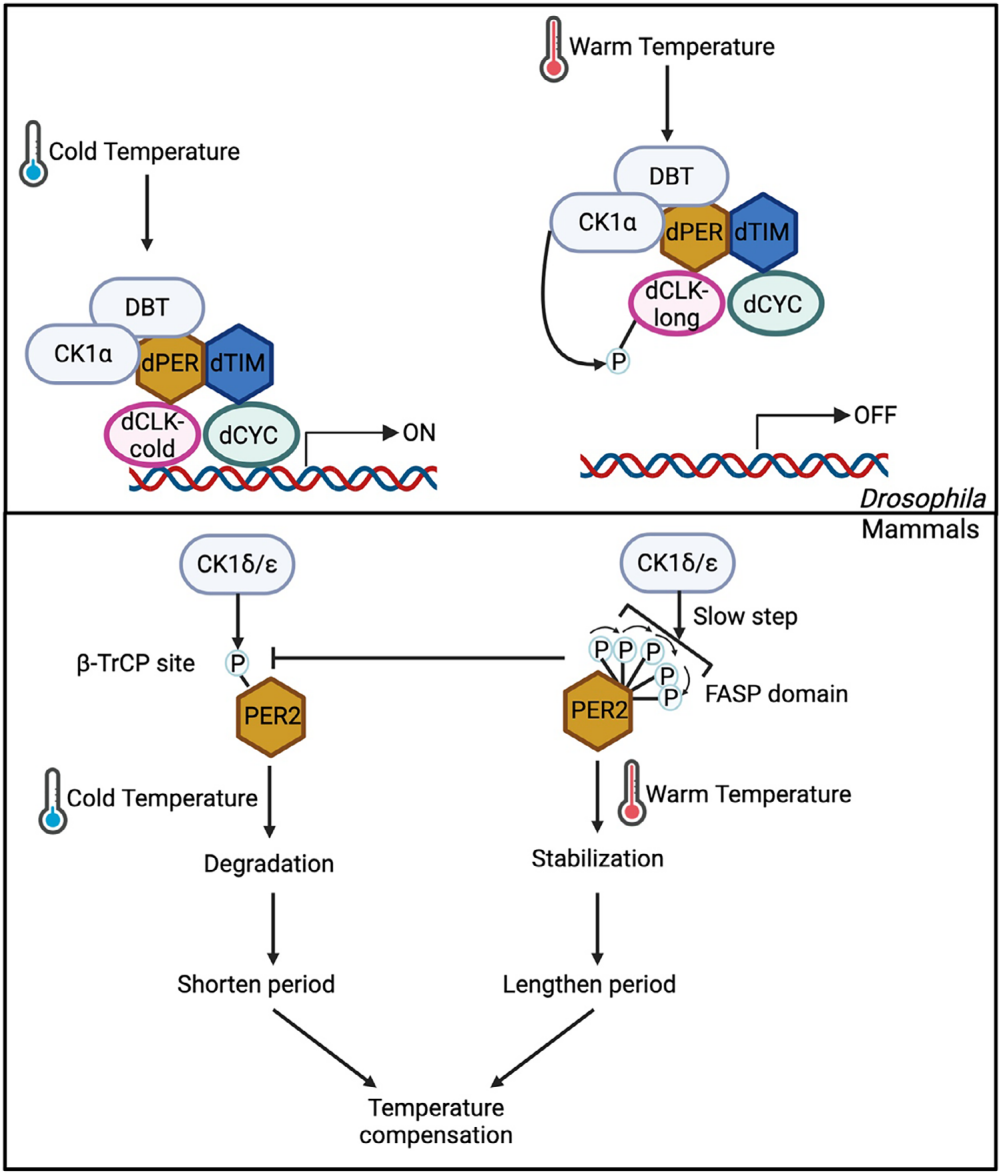
Regulation of clock proteins by temperature through PTM mechanisms. In *Drosophila*, dclk mRNA is alternatively spliced into dclk‐cold under cold conditions. Because dCLK‐cold lacks the CK1α‐targeted phosphosite, it can bind to DNA and activate the transcription of downstream genes. At warm temperatures, dCLK‐long is phosphorylated by CK1α, which prevents its DNA binding and transcriptional activation. In mammals, the “phosphoswitch” model of PER2 provides a molecular basis for temperature compensation. Under cold conditions, CK1δ/ε preferentially phosphorylate the β‐TrCP recognition site on PER2, promoting PER2 degradation and shortening the circadian period‐thereby compensating for the overall slowing of biochemical reactions at lower temperatures. Conversely, at warm temperatures, CK1δ/ε preferentially phosphorylate the FASP domain, which occurs more slowly and inhibits phosphorylation at the β‐TrCP site. As a result, PER2 is stabilized, lengthening the circadian period and compensating for the accelerated biochemical processes at higher temperatures. Created with Biorender.com.

Besides light and nutrient, temperature is another important environmental signal to the circadian clock. Temperature affect the molecular oscillator in two ways: 1) daily temperature cycle provide time cues to entrain circadian clock, termed temperature entrainment; 2) the circadian clock maintain 24 h free‐running period in a wide range of temperature, termed temperature compensation [265, 266]. While most studies revealed the mechanisms underlying temperature entrainment at the post‐transcriptional level [267], PTMs have also been implicated in this process. In flies expressing nonphosphorylatable dCLK variants, temperature entrainment is impaired [268]. Another study provides site‐specific insight. Flies expressing nonphosphorylatable dCLK(S13A) variant or phosphomimetic dCLK(S13D) both display temperature entrainment defect [89]. Interestingly, in response to temperature, flies express CLK‐cold due to alternative splicing, which does not contain S13 residue for phosphorylation. This study demonstrates site‐specific phosphorylation of a core clock protein modulates the response of circadian clocks to temperature changes (Figure 10, Table 1 and Table 2).

The “phosphoswitch” model for PER2 degradation has revealed the mechanisms underlying temperature compensation in mammalian clocks [37] (Figure 10, Table 1). Early studies found that CK1ε/δ‐dependent phosphorylation and PER2 degradation are insensitive to temperature changes, and mutations of CK1ε in hamster and mouse affect their temperature sensitivity of molecular oscillators [158, 248, 269]. In the study of three‐stage degradation of PER2, Zhou et al. [37] demonstrated that under warmer temperature (37°C) the FASP domain is phosphorylated by CK1ε and thus slowing down the degradation of PER2. Whereas at lower temperature (30°C), the β‐TrCP site is preferentially phosphorylated and speeds up the degradation of PER2. This model is highly consistent with the later studies. Structural studies of CK1δ have further identified two potential mechanisms underlying temperature compensation: 1) at low temperature, CK1δ exhibits higher affinity for ATP when mediating a single site phosphorylation (i.e., prefer β‐TrCP site); 2) at high temperatures, CK1δ shows higher affinity for ADP when catalyzing multiple‐site phosphorylation (i.e., prefer FASP domain). Indeed, the mutation of β‐TrCP site on PER2 impairs the temperature compensation in mouse embryonic fibroblasts [175]. Philpott et al. [176] showed that phosphorylation at FASP domain inhibits CK1 activity by binding to its active site, which provides an explanation high temperature slows down PER2 degradation. Moreover, Joshi et al. showed that the phosphorylation of per‐short domain and S47 site is essential for the temperature compensation of fly clock [270]. Findings in mammals and flies suggest that “phosphoswitch” model and the corresponding kinase are conserved features of temperature compensation in mammalian and *Drosophila* clocks.

In summary, the “phosphoswitch” model provided indepth insights for the molecular mechanisms of temperature compensation in both flies and mammals (Figure 10, Table 1 and Table 2). However, organisms experience wider range of daily temperature changes. How PTMs mediate the response of circadian clocks to natural conditions remain unclear. This will shed light on organismal adaptation in natural conditions, especially among seasons.

### 3.4 Other Cellular and Physiological Conditions

Despite aforementioned PTMs promote the robustness and timekeeping of the molecular oscillator, molecular oscillators can still be disrupted under certain cellular and physiological conditions. Strong evidence have supported the link of clock disruption to pathogenic processes. In this section, we summarize known PTM mechanisms that mediate the impact of cellular and physiological conditions on the molecular oscillator.

In mammalian tissue culture system, serum shock is a common method to reset the molecular oscillator as it induces the expression of *Per1*. This effect is mediated by Ca^2+^‐dependent PKCα and PKCγ, which phosphorylates CLOCK and promote its nuclear entry [51]. Under inflammatory conditions, Tumor necrosis factor‐α (TNF‐α), a proinflammatory cytokine, stabilizes CRY1 via upregulating USP2a [196]. Under critical oxidative stress induced by near‐lethal dosage of H_2_O_2_, CK2 phosphorylates BMAL1 at residue S90 to trigger clock resetting [48]. Osmotic stress has been shown to alter the period length and phase of PER2::LUC in both cell culture and SCN slice. The apoptosis signal‐regulating kinase (ASK) mediates the impact of osmotic stress by activating mammalian Target of rapamycin (mTOR)‐AKT signaling to phosphorylate CLOCK at S845 [271, 272].

At the organismal level, aging has been shown to disrupt the expression of clock genes [273, 274, 275]. The decline of NAD^+^ metabolism and SIRT1 mediates the impact of aging on circadian clocks, as SIRT1 regulates the acetylation of BMAL1 and PER2 [191, 276]. Importantly, supplement of nicotinamide riboside (NR), the NAD^+^ precursor, in 22 month‐old mice, restore the robustness of circadian clocks [191]. In aortas, aging causes a phase delay of *Per2* expression, which is related to the reduced NO level and decreased BMAL1 S‐nitrosylation [67].

**FIGURE 11:**
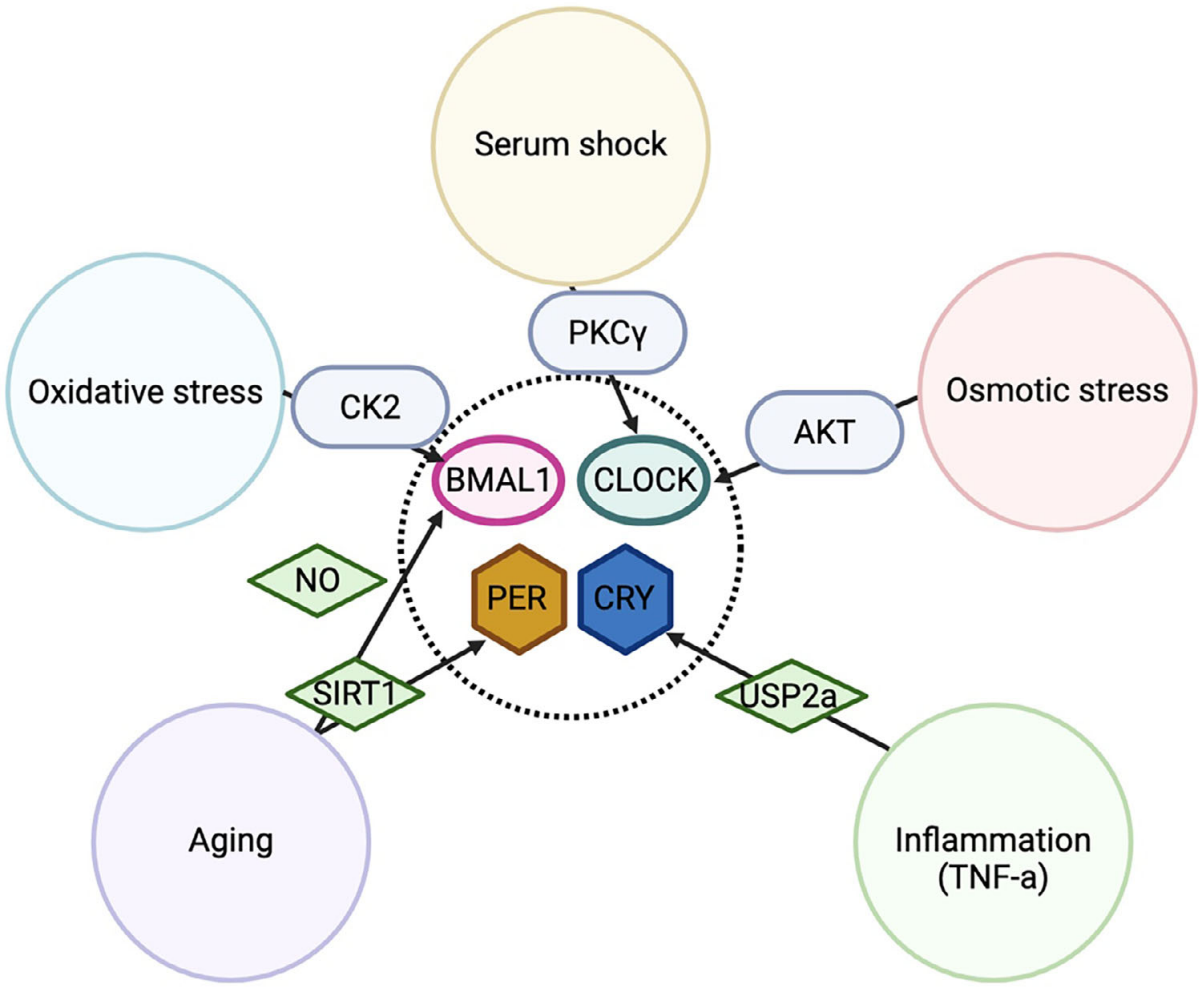
Regulation of circadian clocks by cellular and physiological conditions through PTM mechanisms. The circadian clock responds to a wide range of cellular and physiological cues, including serum shock, osmotic stress, inflammation, aging, and oxidative stress, through diverse PTM‐mediated signaling pathways. Created with Biorender.com.

Taken together, multiple cellular and physiological conditions modulate the molecular oscillator through PTMs, suggesting the interface of circadian clocks and a broad range of (patho)physiological processes (Figure 11, Table 1). Future studies should aim to delineate the integrative signaling networks linking cellular stress, metabolic rewiring, and PTMs of clock proteins, and explore whether targeted modulation of these pathways can preserve circadian integrity during aging and disease progression.

## 4 Conclusion and Future Perspectives

In the past three decades, the PTMs of clock proteins has been elucidated as essential mechanisms for maintaining the 24 h period of the circadian clock (Figures 2, 3, 4, 5, 6, 7, Table 1 and Table 2). Among all the clock related PTMs, phosphorylation is most well studied. Although the phosphorylation sites of clock proteins are not conserved between flies and mice, the function of several kinases is conserved (Figures 2, 3, 4, 5, 6, 7, Table 1 and Table 2). Proteome‐level analysis suggests enrichment of phosphorylation in short‐disordered regions [277], such as FASP domain and per‐short region. To what extent disordered regions affects circadian timekeeping remains to be investigated [278]. Further investigation is also needed to integrate the function of additional PTMs into the current framework to gain a more comprehensive understanding of the regulation of molecular oscillators. These PTMs include both those already shown to regulate clock proteins (O‐GlcNAcylation [69, 189, 206, 207, 260], acetylation [99, 100, 101, 102, 190, 191], S‐palmitoylation [68], and S‐nitrosylation [67]) and newly discovered modifications (pyrophosphorylation [279] and lactylation [280]). Moreover, beyond the molecular oscillator, emerging studies have revealed daily rhythms of PTMs at the proteomic level, as well as how these modifications respond to environmental signals. In the future, the rapid advancement of mass spectrometry [281, 282, 283, 284, 285] and the ability of AlphaFold to predict PTMs functions [277, 286, 287] will greatly facilitate research into PTM mediated circadian regulation.

With the growing understanding of the post‐translational regulation of the circadian clock, the cooperative interactions between different PTMs, either occurring at distinct sites or involving various types of modifications, have gained increasing recognition in recent years. Mathematical modeling has further enhanced our recognition of the importance of these PTM interactions in ensuring precise timekeeping. More importantly, as exemplified by the “phosphoswitch” regulation of PER proteins in temperature compensation [37, 175, 181] (Figure 10, Table 1 and Table 2), the interaction of PTMs could underline the mechanism by which the circadian clock response to external signals. Given some PTMs in the circadian clock is known to be sensitive to metabolism or aging (Figure 9 and 11, Table 1 and Table 2), it will be interesting to investigate how different PTMs interact, whether these interactions respond to different environmental and physiological conditions and whether the PTMs integrate multiple external signals through their interaction.

Finally, emerging evidence indicates that PTMs of clock proteins can regulate their noncircadian functions beyond TTFLs [53, 288, 289, 290]. For instance, ATM‐mediated phosphorylation of BMAL1 S183 drives BMAL1 localization to DNA damage sites in cancer cells, where it promotes homologous recombination [290]. The phosphorylation of CLOCK S106 by CK2α triggers nuclear export to facilitate cancer cell proliferation by CLOCK‐dependent acetylation of PRPS1/2 [53]. Given these findings, investigating the post‐translational regulation of clock protein beyond TTFL‐particularly under disease conditions‐represents a compelling and important direction for future research.

## Author Contributions

Y.Z. conceived the project. X.L. constructed the outline and drafted this manuscript. Y.Z. and X.L. reviewed and edited the manuscript. X.L. and Y.Z. provided funds for this manuscript. All authors read and approved of the final manuscript.

## Conflicts of Interest

The authors declare no conflicts of interest.

## Data Availability

The authors have nothing to report.
